# Deciphering fucosylated protein-linked *O*-glycans in oral *Tannerella serpentiformis*: Insights from NMR spectroscopy and glycoproteomics

**DOI:** 10.1093/glycob/cwae072

**Published:** 2024-09-19

**Authors:** Stephanie Walcher, Fiona F Hager-Mair, Johannes Stadlmann, Hanspeter Kählig, Christina Schäffer

**Affiliations:** Institute of Biochemistry, Department of Chemistry, University of Natural Resources and Life Sciences, Muthgasse 18, Vienna 1190, Austria; Institute of Biochemistry, Department of Chemistry, University of Natural Resources and Life Sciences, Muthgasse 18, Vienna 1190, Austria; Institute of Biochemistry, Department of Chemistry, University of Natural Resources and Life Sciences, Muthgasse 18, Vienna 1190, Austria; Department of Organic Chemistry, Faculty of Chemistry, University of Vienna, Währinger Straβe 38, Vienna 1090, Austria; Institute of Biochemistry, Department of Chemistry, University of Natural Resources and Life Sciences, Muthgasse 18, Vienna 1190, Austria

**Keywords:** glycoforms, oligosaccharide structure, oral microbiome, protein *O*-glycosylation, S-layer

## Abstract

*Tannerella serpentiformis* is a health-associated Gram-negative oral anaerobe, while its closest phylogenetic relative is the periodontal pathogen *Tannerella forsythia.* The pathogen employs glycan mimicry through protein *O*-glycosylation, displaying a terminal nonulosonic acid aiding in evasion of host immune recognition. Like *T. forsythia*, *T. serpentiformis* cells are covered with a 2D-crystalline S-layer composed of two abundant S-layer glycoproteins–TssA and TssB. In this study, we elucidated the structure of the *O*-linked glycans of *T. serpentiformis* using 1D and 2D NMR spectroscopy analyzing S-layer glycopeptides and β-eliminated glycans. We found that *T. serpentiformis* produces two highly fucosylated, branched glycoforms carrying non-carbohydrate modifications, with the structure [2-*O*Me-Fuc-(α1,2)]-4-*O*Me-Glc-(β1,3)-[Fuc-(α1,4)]-2-NAc-GlcA-(β1,4)-[3-NH_2_, 2,4-*O*Me-Fuc-(α1,3)]-Fuc-(α1,4)-Xyl-(β1,4)-[3-*O*Me-Fuc-(α1,3)]-GlcA-(α1,2)-[Rha-(α1,4]-Gal, where the 3*O*Me-Fuc is variable; each glycoform contains a rare 2,4-methoxy, 3-amino-modified fucose. These glycoforms support the hypothesis that nonulosonic acid is a hallmark of pathogenic *Tannerella* species. A combined glycoproteomics and bioinformatics approach identified multiple sites within TssA (14 sites) and TssB (21 sites) to be *O*-glycosylated. LC-MS/MS confirmed the presence of the *Bacteroidetes O*-glycosylation motif (D)(S/T) (L/V/T/A/I) in *Tannerella* species, including the newly identified candidate “N” for the third position. Alphfold2 models of the S-layer glycoproteins were created revealing an almost uniform spatial distribution of the two glycoforms at the N-terminal two thirds of the proteins supported by glycoproteomics, with glycans facing outward. Glycoproteomics identified 921 unique glycopeptide sequences corresponding to 303 unique UniProt IDs. GO-term enrichment analysis *versus* the entire *T. serpentiformis* proteome classified these proteins as mainly membrane and cell periphery-associated glycoproteins, supporting a general protein *O*-glycosylation system in *T. serpentiformis*.

## Introduction

The human oral microbiome hosts a diverse biofilm community, where interactions are carefully balanced by synergistic and antagonistic effects among bacteria as well as bacteria and host tissues and immune cells (Bloch et al. 2017; Bloch et al. 2019; Kendlbacher et al. 2023), naturally favoring oral health. Tipping this fragile balance can lead to persistent infections like periodontitis (Ebersole et al. 2016; Marsh and Zaura 2017; Naginyte et al. 2019)—a chronic inflammation of the gums (Kinane et al. 2017) which can also be associated with inflammatory pathologies at distant sites and systemic diseases (Hajishengallis 2015; Dominy et al. 2019; Park et al. 2019).

Periodontitis is orchestrated within the oral biofilm, notably by the “red complex” of Gram-negative anaerobic pathogens, comprising *Porphyromonas gingivalis*, *Treponema denticola*, and *Tannerella forsythia* (Hajishengallis 2014; Hajishengallis 2015; Lamont and Hajishengallis 2015). Paradoxically, while the red-complex members use inflammation as an effective tool to procure nutrients from tissue break-down products and to conquer the human oral microflora and beyond, at the same time, they must evade host immune surveillance. *T. forsythia* evades detection by employment of molecular mimicry through glycosylation of its unique cell surface (S-) layer, which covers the bacterium’s entire surface in a 2D crystalline lattice constituted by alternating, heavily glycosylated S-layer proteins-TfsA and TfsB (Posch et al. 2012; Sekot et al. 2012). The S-layer bound *O*-glycan is a complex nonasaccharide that terminates strain-specifically with a modified nonulosonic acid (pseudaminic or legionaminic acid) (Posch et al. 2011; Friedrich et al. 2017), a mimic of human sialic acid, aiding in disguising the bacterium as “self” and ensuring its persistence in the host (Settem et al. 2013; Tomek et al. 2018). This *O*-glycan abundantly decorates the *T. forsythia* S-layer proteins at Ser or Thr residues within the predicted phylum-wide three amino-acid motif (D)(S/T)(A/I/L/V/M/T/S/C/G/F) (Posch et al. 2011; Coyne et al. 2013; Veith et al. 2021). Notably, in *T. forsythia*, also several other proteins carry this *O*-glycan, arguing for the presence of a general protein *O*-glycosylation system (Posch et al. 2011; Veith et al. 2021). Very recently, several new candidates for the third position in the glycosylation motif were identified in *Prevotella intermedia,* another member of the *Bacteroidetes* phylum, namely (N/E/Q/D/P), summing up to a total of 15 candidates for the third position (Ye et al. 2024). The closest known phylogenetic relative of *T. forsythia*–the Gram-negative, anaerobic oral bacterium *Tannerella serpentiformis* (previously, *Tannerella* HOT-286*;* phylotype BU036)—is considered periodontal health-associated (Frey et al. 2018; Ansbro et al. 2020). This is supported by the absence of several virulence-associated genes in comparison to the pathogenic *Tannerella* species, encoding *e.g*., surface antigens (Sharma et al. 1998; Onishi et al. 2008; Beall et al. 2018) and enzymes (Stafford et al. 2012; Beall et al. 2018). Furthermore, the bacterium has recently been shown to practice a less invasive lifestyle compared to *T. forsythia* and to mitigate growth and biofilm formation of five other oral pathogens, including *T. forsythia* and *P. gingivalis*, when studied in a polymicrobial biofilm model (Kendlbacher et al. 2023). The surface of *T. serpentiformis* cells is covered by an S-layer that resembles that of *T. forsythia* and is composed of two high-molecular weight glycoproteins, according to a carbohydrate-positive stained SDS-PAGE of bacterial cells, supported by electron microscopy (Frey et al. 2018; Kendlbacher et al. 2023). TssA and TssB, the S-layer proteins of *T. serpentiformis*, share 52% and 59% amino acid identity, respectively, with the corresponding S-layer proteins TfsA (UniProt A0A1D3UND3) and TfsB (UniProt A0A1D3UN43) of *T. forsythia*, and antibodies against *T. forsythia* S-layer proteins do not recognize those of *T. serpentiformis* (Kendlbacher et al. 2023).

This study aims to elucidate the composition and structure of the *T. serpentiformis O*-glycan and its linkage to the bacterium’s abundant S-layer proteins, driven by the hypothesis that an altered, nonulosonic acid-free glycan contributes to the health-associated lifestyle of this *Tannerella* species. To achieve this, proteins were isolated from whole bacterial cells grown anaerobically on blood agar plates, followed by proteolytic digestion and purification of glycopeptides. NMR spectroscopy, in conjunction with MS analysis of glycopeptides and β-eliminated glycan samples, revealed two similar yet distinct (un)decasaccharide species, which correspond to complex, heavily fucosylated and modified *O*-glycans. Furthermore, this study investigated and confirmed the predicted phylum-wide three-amino-acid protein *O*-glycosylation motif at Ser and Thr sites, based on accumulated MS data, notably, revealing “N” as a novel amino acid at position 3.

## Results

### Glycan structure elucidation by NMR spectroscopy

To elucidate the structure of the *O*-glycan from *T. serpentiformis*, two types of NMR samples were analyzed-β-eliminated glycan preparations and purified glycopeptides.

**Fig. 1:**
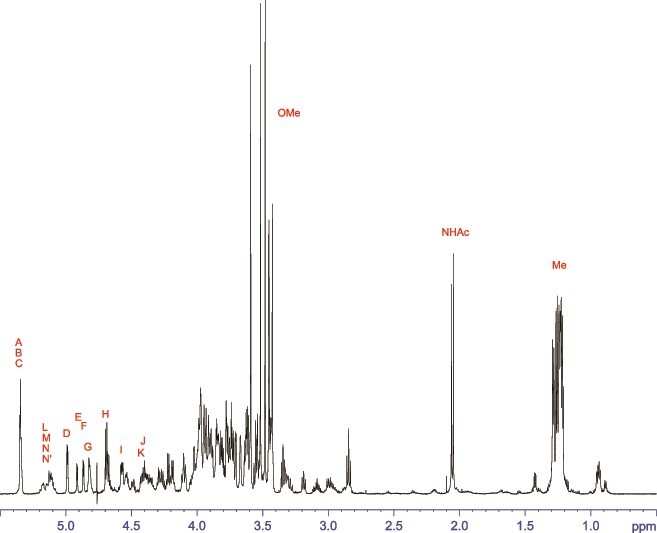
^1^H NMR spectrum of the purified protein-linked *O*-glycan from *T. serpentiformis.*

**Fig. 2:**
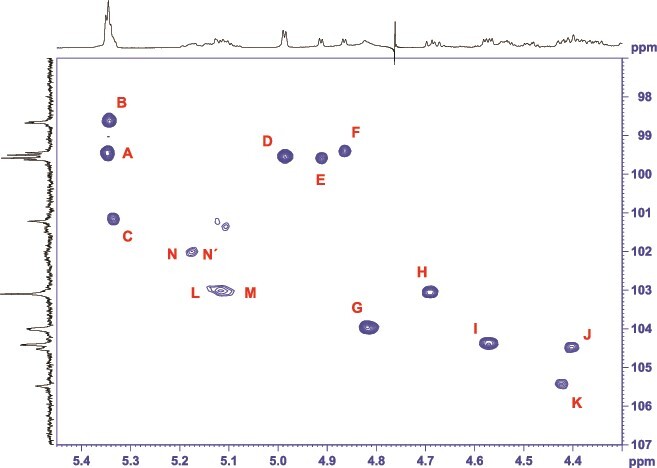
HSQC spectrum of the purified protein-linked *O*-glycan from *T. serpentiformis*, showing the anomeric region. Top trace, ^1^H NMR spectrum; left trace, ^13^C NMR spectrum.

The ^1^H NMR spectrum (Fig. 1) of the purified glycopeptide sample dissolved in D_2_O exhibited expected signals in the carbohydrate chemical shift range, revealing several doublets at 1.25 ppm, indicative of methyl groups of 6-deoxy sugars. Additionally, the glycopeptide displayed significant methylation, as evidenced by sharp signals between 3.4 and 3.6 ppm. In total, six resonances could be assigned to methoxy groups, with three signals appearing to have half the intensity of the others. Anomeric information, depicted in the appropriate region of an HSQC spectrum (Fig. 2), revealed varying intensities among individual anomeric protons. Additionally, duplication of certain anomeric signals was observed on both proton and/or carbon frequencies, while other anomeric signals (Fig. 2, ^1^H chemical shift range between 5.05 and 5.20 ppm) exhibited significant overlap by multiple lines. Thus, the initial examination of the NMR spectra suggested a nonhomogeneous structure in both the glycan and the peptide portion of the purified peptide-linked *O*-glycan.

Subsequently, a comprehensive NMR analysis, employing a range of different 1D and 2D NMR spectra was focused on the identification of the individual monosaccharides, taking the anomeric signals as starting points. The entire spin systems could be derived from 2D-TOCSY traces, or 1D-TOCSY spectra by selective excitation of one uncovered signal or from DREAMTIME 1D-TOCSY spectra (Jenne et al. 2022) by selective excitation of two spin-coupled protons simultaneously. All the ^1^H NMR assignments were confirmed through spin simulation. The chemical shifts, *J* couplings, and line width of the individual signals were adjusted until the calculated spectrum matched the experimental one.

The most deshielded ^1^H NMR signal (as per the annotation of the anomeric signals in Fig. 2) represents an overlay of three anomeric protons, labeled as **A**, **B**, and **C**. Their TOCSY spin systems terminate at position 4, displaying a proton signal with minimal *J*-coupling (Supplementary Fig. S1), indicative of a part of a galactose-type unit. A strong NOESY cross-peak from these protons 4 to methyl groups in the aliphatic region (Supplementary Fig. S2) identified all three monosaccharides as fucoses. The missing protons 5 were identified in the DQF-COSY spectrum by their corresponding cross-peaks to the methyl groups (Supplementary Fig. S3). Sugar **C** exhibits only half the intensity compared to sugar **A** or **B**. In addition, all three are methylated, confirmed by HMBC ^1^H-^13^C long range cross-peaks between the methyl protons and the carbon at the linkage site, with **A** at position 2, **C** at position 3, and **B** being even dimethylated at positions 2 and 4 (Supplementary Fig. S4). Once more, the methoxy group C3 displays half the intensity compared to the others. In unit **B**, the methoxy group 2 exhibits two lines, indicating variability in the glycan chain. These findings suggest two proposed glycoforms that differ by the presence or absence of fucose **C**. Further investigation of the ^13^C NMR shifts of fucose **B** revealed another unusual detail: carbon 3 exhibits a chemical shift of 53.9 ppm, indicating that this monosaccharide is a 3-amino-3-deoxy-α-fucopyranose. Moving forward, the three isolated anomeric protons, labeled **D**, **E**, and **F**, were also assigned to fucoses (Supplementary Fig. S5), with the spin systems for **E** and **F** being highly similar. Given their reduced intensities compared to **D**, such as the anomeric protons (Figs 1 and 2) or the methyl carbons 6 (Supplementary Fig. S6), it is conceivable that **E** and **F** belong to different glycoforms. So, the glycopeptide is highly fucosylated with either four or five units, and all of them are α-pyranoses. Continuing with unit **G**, this spin system includes the last remaining aliphatic methyl group, indicating a 6-deoxysugar, specifically an α-rhamnose. Since the anomeric ^1^H NMR signal spans a broader chemical shift range due to the overlap of several lines, this monosaccharide is likely located proximal to the inhomogeneous peptide portion. Subsequently, the next spin systems in sequence are two β-glucose-type sugars. Unit **H** was identified as β-glucopyranose, methylated at position 4, while sugar **I** was determined to be 2-acetamido-2-deoxy-β-glucuronic acid (Supplementary Fig. S7). Finally, sugars **J** and **K** were identified as pentoses, two β-xylopyranoses (Supplementary Fig. S8), with one unit for each of the two glycoforms. The remaining unassigned anomeric cross-peaks, labelled **L**, **M**, and **N** (Fig. 2), exhibit a very complex pattern spread over an extended chemical shift range on the proton axis; the signals on the carbon axis are similarly expanded, resulting in individual resonance lines with very poor signal-to-noise ratio. Consequently, the corresponding spin systems in the conventional spectra derived for these units are diffuse and could not be unambiguously attributed to a specific monosaccharide. It is presumed that these sugars are located near the reducing end of the glycan and these signals essentially reflect the inhomogeneity of the peptide portion, as variations in the amino acid sequence yield diverging chemical shifts for the nearby linked sugars.

To simplify the complexity of the afore mentioned resonances, a β-eliminated glycan sample was prepared. With the absence of the peptide portion in this sample, the associated variability in the spectra was completely eliminated, particularly for the sugars near the reducing end of the glycan. However, simultaneously, the identity of the terminal sugar was lost, as reductive β-elimination results in the corresponding sugar alcohol, which cannot be determined by NMR in relation to the original monosaccharide anymore. In the HSQC spectrum of the β-eliminated glycan (Supplementary Fig. S9), the anomeric region displayed two sharp doublets on the proton frequency for proton 1 of unit **L** and **M**, resonating close together, yielding a triplet-like signal. Additionally, α-rhamnopyranose **G**, which previously exhibited an extended anomeric signal due to variations in the peptide part of the intact glycopeptide, now presented two sharp resonances with a very small, nearly unresolved *J*-coupling due to the α-*manno* configuration (Supplementary Fig. S10). The identification of the spin systems for sugars **L** and **M** was straightforward, indicating an α-glucuronic acid; this result could be correlated to the glycopeptide using the DREAMTIME selection scheme. Exciting protons 4 and 5 simultaneously, which share a relatively large *J*-coupling of 9.5 Hz, followed by a TOCSY spin-lock, facilitated their assignment also in the intact preparation (Supplementary Fig. S11). The derived chemical shifts for **L** and **M** are notably different, distinguishing them as the potential linkage point for the 3-*O*-methyl-β-fucopyranose **C**, which is either 1,3 linked to α-glucuronic acid **M** in one glycoform or is absent in the other, thus lacking substitution on unit **L**.

Finally, to analyze the remaining unassigned monosaccharide at the reducing end of the glycan linked to the peptide portion, the spectra of the intact glycopeptide preparation were reexamined. Within the region of or close to the broad and low-intensity envelope of overlapping anomeric signals (Fig. 2) of the previously identified α-glucuronic acid **L/M**, additional extended cross-peaks with very low intensity are visible, denoted as **N/N´**. Several attempts were made to select the associated spin systems using the DREAMTIME-TOCSY approach. The only feasible option was to utilize the spin pairs proton 1 and 2, thereby obtaining the chemical shift for H2 from the corresponding COSY cross-peak, which is very weak, almost at noise level. Since the involved *J*-coupling between protons 1 and 2 is small due to the α-configuration of the unknown sugar, the evolution time of the antiphase signal is prolonged, reducing the transfer efficiency for the double quantum coherence. Consequently, the subsequent TOCSY transfer yields spectra with very low signal-to-noise ration even after extended experiment times. Eventually, a spin system with four protons was generated, with proton 4 exhibiting only a very small *J*-coupling, indicative of to a galactose-type sugar (Supplementary Fig. S12). Unfortunately, due to signals extending over a large chemical shift range, low intensity, and severe signal overlap, not all proton and carbon chemical shifts for the different variations could be assigned. Nevertheless, by combining all NMR results and supported by mass spectrometry (see below), the linkage sugar to the peptide portion could be identified as α-galactose. Detailed NMR information for the peptide portion could not be extracted due to the high variability in the glycosylation amino acid motif.

All NMR data for the individual monosaccharides are compiled in Table 1. The anomeric configuration was established either by analyzing the *J*-coupling between proton 1 and 2 or by the one-bond ^1^H-^13^C J-coupling derived from an HSQC spectrum without ^13^C decoupling. Supported by HMBC cross-peaks between positions 1 and 5, all sugar units could be identified as pyranoses.

**Table 1:** 

a) ^1^H and ^13^C chemical shifts (ppm) and in parenthesis *J* couplings (Hz) of the purified protein-linked *T. serpentiformis O-*glycan.								
Chemical shift δ ^1^H (J_HH_)^a^ and ^13^C (J_CH_)								
Unit	1	2	3	4	5	6	other	
**A**	5.350	3.438	3.633 / 3.629^b^	3.706	4.218	1.285 / 1.283^b^	2-OMe 3.480	
α-(2-*O*Me)-Fuc*p*	(3.8)	(3.8 / 10.5)	(10.5 / 3.0)	(3.0 / 1.0)	(1.0 / 6.5)	(6.5)		
	99.51 (172.7)	80.22	70.99	74.40	68.93	18.35	60.28	
**B**	5.346 / 5.344^b^	3.535	3.619	3.668	4.186	1.219	2-OMe 3.454 /	4-OMe 3.590
α-(2,4-*O*Me)-(3-NH_2_)-Fuc*p*	(3.4)	(3.4 / 11.0)	(11.0 / 2.9)	(2.9 / 1.3)	1.3 / 6.6)	(6.6)	3.451^b^	
	98.68 (173.9) / 98.65^b^	76.52	53.91	81.30	69.05	17.95	59.91	64.63
**C**	5.338 / 5.333^b^	3.808	3.543	4.023	4.541	1.212	3-OMe 3.426	
α-(3-*O*Me)-Fuc*p*	(4.0)	(4.0 / 10.3)	(10.3 / 2.8)	(2.8 / 1.0)	(1.0 / 6.4)	(6.4)		
	101.22 (173.2)	69.79	81.46	70.31	69.07	18.13	58.75	
**D**	4.987	3.728	3.940	3.776	4.677	1.259		
α-Fuc*p*	(3.9)	(3.9 / 10.3)	(10.3 / 3.2)	(3.2 / 1.0)	(1.0 / 6.7)	(6.7)		
	99.58 (172.1)	70.17	71.77	74.66	69.42	18.23		
**E**	4.913	3.809	3.979	3.950	4.289	1.246		
α-Fuc*p*	(3.9)	(3.9 / 10.6)	(10.6 / 2.8)	(2.8 / 1.2)	(1.2 / 6.5)	(6.5)		
	99.63 (171.0)	70.18	75.87	80.43	69.55	18.59		
**F**	4.865 (3.9)	3.799	3.977	3.944	4.264	1.235		
α-Fuc*p*	(3.9)	(3.9 / 10.6)	(10.6 / 2.8)	(2.8 / 1.2)	(1.2 / 6.5)	(6.5)		
	99.44 (172.1)	70.18	75.97	80.49	69.44	18.61		
**G**	4.819	3.975	3.732	3.437	3.992	1.232		
α-Rha*p*	103.99 (169.9) / 104.01^b^	73.07	72.94	74.64	71.75	19.14		
**H**	4.692	3.345	3.768 / 3.765^b^	2.843	3.434	3.920	4-OMe 3.515	
β-(4-*O*Me)-Glc*p*	(8.0)	(8.0 / 9.3)	(9.3 / 8.8)	(8.8 / 9.8)	(9.8 / 2.0 / 7.6)	(11.4 / 2.0)		
						3.611		
						(11.4 / 7.6)		
	103.10 (163.9)	80.87	79.15	83.80	77.81	64.13	63.08	
**I**	4.579 / 4.573^b^	3.929 / 3.926^b^	4.105 / 4.104^b^	3.900 / 3.896^b^	3.849 / 3.844^b^			CH_3_ 2.060 / 2.045^b^
β-Glc*p*ANAc	(8.0)	(8.0 / 10.1)	(10.1 / 9.5)	(9.5 / 9.4)	(9.4)			
	104.42 (161.2) / 104.40^b^	58.04	77.09	76.52	79.00	176.51	C=O 177.14 /	24.86 /
							177.11^b^	24.84^b^
**J**	4.405	3.187	3.552	3.497	4.044			
β-Xyl*p*	(7.7)	(7.7 / 9.4)	(9.4 / 9.2)	(9.2 / 5.7 / 10.1)	(11.6 / 5.7			
					3.289			
					(11.6 / 10.1)			
	104.54 (162.9)	76.21	76.37	76.60	65.04			
**K**	4.425	3.317	3.553	3.606	4.093			
β-Xyl*p*	(7.7)	(7.7 / 9.4)	(9.4 / 9.2)	(9.2 / 5.7 / 10.1)	(11.6 / 5.7)			
					3.325			
					(11.6 / 10.1)			
	105.48 (163.9)	75.68	76.49	76.71	65.19			
**L**	5.133	3.632	3.844	3.742	4.352			
α-Glc*p*A	103.02 (172.4)	74.09	73.75	82.16	73.66	176.68		
**M**	5.107	3.844	3.975	3.902	4.379			
α-Glc*p*A	103.08 (172.4)	75.11	77.31	77.76	74.45	176.68		
**N**	5.177	3.900	4.031	3.895	3.625	3.823 /3747		
α-Gal*p*	102.06 (171.9)	83.46	71.58	78.38	75.01	63.03		
**N´**	5.171	3.732	3.611	3.708	n.a.	n.a.		
α-Gal*p*	102.06 (171.9)	82.19	74.99	78.26				
**A**	5.351 (3.8)	3.434	3.638	3.714	4.216	1.289	2-OMe 3.480	
α-(2-*O*Me)-Fuc*p*	99.47 (171.6)	80.23	70.96	74.40	69.00	18.40	60.27	
**B**	5.325	3.486	3.590	3.683	4.216	1.211	2-OMe 3.437	4-OMe 3.567
α-(2,4-*O*Me)-(3-NH_2_)-Fuc*p*	98.83 (172.4)	77.04	53.89	81.61	69.06	17.98	59.94 / 59.95^d^	64.63
**C**	5.331 (4.0)	3.784	3.580	4.026	4.551	1.207	3-OMe 3.426	
α-(3-*O*Me)-Fuc*p*	101.07 (173.4)	69.94	81.46	70.36	68.94	18.14	58.75	
**D**	5.043	3.736	3.929	3.775	4.686	1.259		
α-Fuc*p*	99.47 (171.0)	70.19	71.85	74.70	69.25	18.24		
**E**	4.917 (3.9)	3.802	3.969	3.928	4.261	1.243		
α-Fuc*p*	99.63 (170.9)	70.22	76.10	80.67	69.45	18.66		
**F**	4.866 (3.9)	3.794	3.964	3.921	4.286	1.253		
α-Fuc*p*	99.47 (171.4)	70.09	76.17	80.67	69.54	18.67		
**G**	4.971 / 4.953^d^	4.018 / 4.014^d^	3.808 / 3.804^d^	3.454 / 3.452^d^	3.793 / 3.789^d^	1.274 / 1.269^d^		
α-Rha*p*	(1.4)	(1.4 / 3.1)	(3.1 / 9.8)	(9.8 / 9.5)	(9.5 / 6.2)	(6.2)		
	104.80 (168.5) / 104.82^d^	73.35	72.61	74.62	70.19	19.36 / 19.33^d^		
**H**	4.693	3.349	3.768	2.838	3.435	3.917	4-OMe 3.515	
β-(4-*O*Me)-Glc*p*	(8.0)	(8.0 / 9.3)	(9.3 / 8.8)	(8.8 / 9.8)	(9.8 / 2.0 / 7.6)	(11.4 / 2.0)		
						*3.612*		
						(11.4 / 7.6)		
	103.10 (161.4)	80.85	79.17	83.79	77.81	64.14	63.05	
**I**	4.551 (7.8) / 4.545^d^	3.912	4.075	3.906	3.741 / 3.738^d^			CH_3_ 2.054 / 2.040^d^
β-Glc*p*ANAc	104.47 (161.0) / 104.44^d^	58.10	77.24	76.70	80.04	177.26	C=O 177.11	24.86
**J**	4.445 (7.7)	3.166	3.565	3.305	4.036 / 3.285			
β-Xyl*p*	104.24 (161.0)	76.37	76.46	76.66	65.07			
**K**	4.422 (7.7)	3.316	3.565	3.602	4.091 / 3.326			
β-Xyl*p*	105.71 (160.7)	75.82	76.46	76.68	65.24			
**L**	5.216	3.597	3.799	3.656	4.200			
α-Glc*p*A	(4.0)	(4.0 / 9.9)	(9.9 / 8.9)	(8.9 / 10.0)	(10.0)			
	102.38 (170.4)	74.02	74.03	83.23	74.64	178.52		
**M**	5.211	3.811	3.953	3.853	4.220			
α-Glc*p*A	(4.0)	(4.0 / 9.9)	(9.9 / 8.9)	(8.9 / 10.0)	(10.0)			
	102.38 (170.4)	75.37	77.47	78.26	75.48	178.32		
Alditol	3.922 / 3.880	3.707	4.017	4.046 / 4.027^d^	n.a.	3.816 / 3.642		
	63.80	82.33 / 82.17^d^	71.17	82.04 / 81.97^d^	n.a.	65.12		

n.a. not assigned.

^a^ *J*_HH_ couplings analyzed by spin-simulation.

^b^additional signals due to heterogeneity of the glycopeptide.

^c^ *J*_HH_ couplings analyzed by spin-simulation.

^d^additional signals due to the two glycoforms of the β-eliminated glycan.

**Fig. 3:**
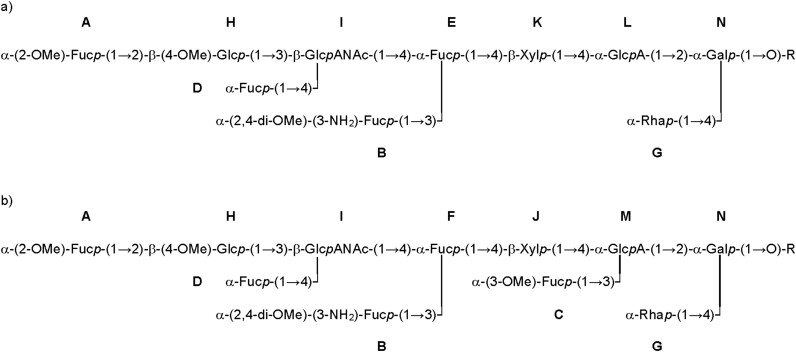
Structure of the two glycoforms of the protein-linked *O*-glycan from *Tannerella serpentiformis*. **A**) Decasaccharide (Glycoform G1), **B**) Undecasaccharide (Glycoform G2). R, peptide portion. WURCS2 string for G2: WURCS = 2.0/10,11,10/[a2112h-1x_1-5][a2211m-1a_1-5][a2122A-1a_1-5][a1221m-1a_1-5_3*OC][a212h-1b_1-5][a1221m-1a_1-5][a1221m-1a_1-5_3*N_2*OC_4*OC][a2122A-1b_1-5_2*NCC/3 = O][a2122h-1b_1-5_4*OC][a1221m-1a_1-5_2*OC]/1-2-3-4-5-6-7-8-9-10-6/a2-b1_a4-c1_c3-d1_c4-e1_e4-f1_f3-g1_f4-h1_h3-i1_h4-k1_i2-j1.

With all spin systems of the building blocks in hand, the glycosidic linkage information was determined using ^1^H-^13^C long-range correlations aided by NOESY information (Supplementary Fig. S2). Supplementary Fig. S13 illustrates the HMBC spectrum with the relevant cross-peaks between the anomeric protons and the corresponding carbons at the linkage position of the following sugars, culminating in the structure of the two glycoforms—one decasaccharide (termed G1) and one undecasaccharide (termed G2) with an additional fucose attached (Fig. 3).

As the glycans are highly branched and fucosylated, the NMR data were further analyzed to identify possible secondary structural elements. Comparing the ^1^H NMR chemical shifts for protons 5 of the fucoses, a noticeable downfield shift to 4.6 ppm for sugar **D**, and a less pronounced shift to 4.54 ppm for sugar **C** was observed, while sugars **A, B, E,** and **F** were in the range of 4.19 and 4.29 ppm. Such a downfield shift of a Fuc H5 appears in branched trisaccharide sequences X-β1,3-[Fucα1,4]-Y and X-β1,4-[Fucα1,3]-Y (Aeschbacher et al. 2017). Here, Y is a saccharide with equatorial hydroxyl groups at positions 3 and 4. The fucose is stacked with sugar X, resulting in a short distance between Fuc H5 and X-O5; the shorter the distance, the higher the resulting downfield shift. The Fucα1,4 motif can be found in the investigated glycans for Fuc **D** (X = Glc **H**, Y = GlcANAc **I**; δ = 4.68 ppm). This stacking is further confirmed by an inter residue NOE contact between the methyl group of Fuc **D** and H2 of glucose **H** (Supplementary Fig. S2). In the G2 undecasaccharide, the motif Fucα1,3 appears for Fuc **C** (X = Xyl **J**, Y = GlcA **M**; δ = 4.54 ppm); a NOESY cross-peak between **C**-H6 and **J**-H2 validates this stacking (Supplementary Fig. S2), although it is less pronounced given the smaller downfield shift of H5 and the weaker NOESY contact compared to the situation for Fuc **D**. Consequently, the flexibility and rigidity of the two glycoforms G1 and G2 seem to differ slightly, which could affect the recognition of individual glycoepitopes.

### Glycoproteomics

**Fig. 4:**
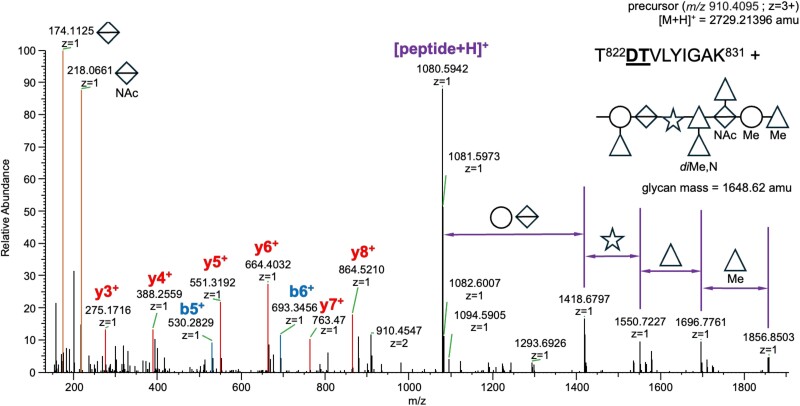
Representative MS/MS spectrum of a *T. serpentiformis* glycopeptide. Higher-energy collisional dissociation (HCD) of multiply charged glycopeptide precursor ions (*m/z *910.4095; charge state 3+) enables the identification of the amino acid sequence of the *O*-linked glycopeptide T822DTVLYIGAK831 (of protein A0A2R4KIF3_9BACT) based on peptide-sequence specific fragment ions (y-ions, b-ions); additionally, it allows for a partial structural/compositional characterization of the glycan moiety. Mass differences between glycopeptide fragments are indicated by the corresponding symbols for the respective monosaccharide classes according to the symbol nomenclature for Glycans (SNFG) (Varki et al. 2015);* i.e.*, hexoses as circles, deoxyhexoses as triangles, hexuronates as divided diamonds, pentoses as stars; Me for methylation, NAc for *N*-acetylation, N for amino modification.

For glycoproteomic analysis, the two S-layer glycoprotein bands from *T. serpentiformis*, TssA (feature BCB71_RS00675 in GenBank: CP017038.2, UniProt A0A2R4KII9) and TssB (feature BCB71_RS00680 in GenBank: CP017038.2, UniProt A0A2R4KIF3) (Kendlbacher et al. 2023), were excised from an SDS-PAGE gel of a whole bacterial cell lysate, tryptically digested in-gel, and analyzed by LC-MS/MS. MS analysis revealed that the excised bands contained, in addition to the expected TssA and TssB glycoproteins (Kendlbacher et al. 2023), nine other co-migrating glycoproteins (see “Data-analysis and glycopeptide identification” in the “Materials and Methods” section and Supplementary Table S1 for a list of identified peptide sequences and their corresponding UniProt IDs). The sample included 22 unique peptide fragments that could be mapped to TssA and 15 unique peptide fragments that could be mapped to TssB. Intrigued by the unexpectedly large number of glycoproteins, which evidentially co-migrated with the S-layer proteins, the full *T. serpentiformis* proteome from a whole-cell lysate was analyzed by LC-MS/MS. This analysis revealed 8,896 MS/MS spectra identified as glycopeptides and could be mapped to 921 unique peptide sequences, corresponding to 303 unique UniProt IDs, with TssA present 99 times and TssB 94 times (see Supplementary Table S2_Skyline-output_Tannerella serpentiformis_Glycoproteome.xlsx). The dataset allowed for an overall estimate of the relative abundance of glycan masses contained in the proteome sample (Supplementary Fig. S17). A representative glycopeptide spectrum is shown in Fig. 4. The two most abundant glycan masses (*i.e.*, 1,634 Da and 1,794 Da) clearly stood out and could be attributed to the G1 and G2 glycoforms of *T. serpentiformis* (compare with Fig. 3, Supplementary Fig. S14). GO-term enrichment analysis versus the entire *T. serpentiformis* proteome classified most of the glycoproteins as (outer) membrane proteins and proteins associated with the cell periphery or cell envelope (Supplementary Fig. S15).

### Glycosylation amino acid motif

**Fig. 5:**
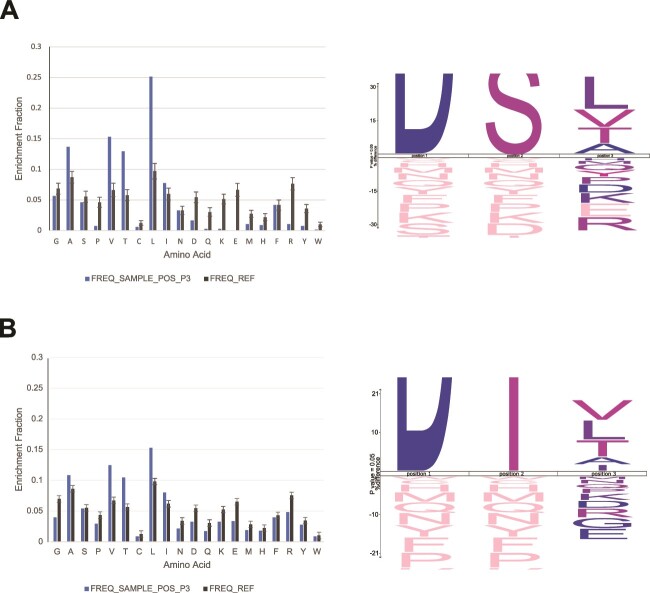
**A**) Left, enriched amino acids in the third position of the amino acid motif (D)(S/T)(X) in glycopeptides derived from whole *T. serpentiformis* cells as determined against an unglycosylated proteome background (UP000240373). Right, general amino acid sequence motif (D)(S/T)(L/V/T/A) based on detected glycopeptides with the motif search D(S/T)(X). **B**) Left, enriched amino acids in the third position of the amino acid motif (D)(S/T)(X) in full glycoprotein sequences retrieved from the proteome UP000240373 of *T. serpentiformis* as determined against an unglycosylated proteome background. Right, general amino acid sequence motif (D)(S/T)(L/V/T/A/I) based on full glycoprotein sequences retrieved from the *T. serpentiformis* proteome (UP000240373) with the motif (D)(S/T)(X). Compared to the glycopeptide search, “I” in third position is now statistically significant. The figure zooms into the third and most variable position to optimize legibility, cutting off the letters of the first and second positions (“D(S/T)”) since these positions are predetermined in the motif. All figures were generated with IceLogo (Colaert et al. 2009).

The NMR analysis has already revealed a high degree of variation in the peptide portion of the *T. serpentiformis* glycopeptide sample. According to the currently identified glycosylation amino acid motif in the phylum *Bacteroidetes*, only position −1 (position 1 in the three-letter amino acid motif) is conserved as asparagine (D), while position +1 (position 3 in the amino acid motif) varies significantly including 15 different candidates to date (Veith et al. 2021; Veith et al. 2022; Ye et al. 2024). The dataset created was utilized for an organism-wide three amino-acid glycosylation motif search using IceLogo (Colaert et al. 2009), a probability-based analysis tool for visualizing consensus sequences in multiple aligned peptide sequences. The reference set was built from the *T. serpentiformis* W11666 proteome (UP000240373), comprising 2,360 entries, minus all glycoprotein UniProt entries identified in this study (leaving 2,106 entries). This resulted in a reference dataset representing only unglycosylated proteins in this bacterium. In the first step, the sample dataset comprised three amino-acid snippets of peptide sequences containing either “S” or “T” in the second position (motif (X)(S/T)(X)). The analysis convincingly confirmed the amino acid “D” in front of “(S/T)” from a statistical point of view, completely independent of a predefined motif, with a rare occurrence of “T” in the first position, as suggested by the currently agreed-upon amino acid motif (Fletcher et al. 2009; Coyne et al. 2013; Veith et al. 2013) (Supplementary Fig. S16). To probe the final position of the glycosylation motif in *T. serpentiformis*, a second sample set was created, defining “D” in first position and “(S/T)” in second position, leaving only the third position as a variable. This analysis showed that the third position is significantly enriched in “(L/V/T/A)” in the sample dataset consisting of detected glycopeptides, as expected. “I” in third position was also enriched in the sample set, but not to a significant degree (Fig. 5A).

Expanding our analysis to the entire glycoprotein sequences, as provided in the *T. serpentiformis* W11666 proteome (UP000240373), rather than solely focusing on their detected glycopeptide fragments, we observed that “(D)/(S/T)” is most frequently followed by “(L/V/T/A/I)” in the third position, with “I” now emerging as a significantly enriched candidate (Fig. 5B).

To validate this approach, we cross-checked it using a publicly available, similarly generated dataset for *T. forsythia* (Veith et al. 2021). The reference dataset consisted of the *T. forsythia* proteome from UniProt (taxon 28112, UP000182057), which comprises 2,705 entries minus all glycoproteins, resulting in 2,605 entries. The sample set was specified either as “(X)(S/T)(X)” (to probe the first position) or as “(D)(S/T)(X)” (to probe the third position). Amino acid groups derived from the identified glycopeptides or the full glycoprotein sequences, as described above. Once again, “D” predominated as the most likely amino acid to occupy the first position in *T. forsythia*, with rare occurrence of “S” when only “(S/T)” were specified in the second position*.* The results for the third position were also highly similar to the those observed for *T. serpentiformis*. When sampling from detected glycopeptides, the third position was predominantly occupied by “(L/A/V/I/T)” (Supplementary Fig. S17), mirroring the findings in the full protein sequence search for *T. serpentiformis*. However, when the entire protein sequences derived from the *T. forsythia* proteome were considered, only “(L/V/I)” remained significant candidates for the third position in the motif (Supplementary Fig. S18). The amino acids “(A/T)” remained enriched in the sample set based on the full protein sequences, but to an insignificant degree. Thus, the statistically significant fundamental candidates for the third position in the *Bacteroidetes* glycosylation amino acid motif remain the same based on both *Tannerella* species, namely “(L/V/T/A/I)”.

### Glycosylation site occupancy

The distribution of the two major glycoforms G1 and G2 across the two *T. serpentiformis* S-layer proteins, TssA (feature BCB71_RS00675 in GenBank: CP017038.2, UniProt A0A2R4KII9) and TssB (feature BCB71_RS00680 in GenBank: CP017038.2, UniProt A0A2R4KIF3), was investigated. Both proteins are known to be heavily glycosylated (Frey et al. 2018; Kendlbacher et al. 2023), and a high number of potential glycosylation sites was predicted for each protein based on the three amino-acid glycosylation motif (D)(S/T)(A/I/L/V/M/T/S/C/G/F). Specifically, there were 15 sites for TssA and 31 sites for TssB, which includes four new, unpredicted sites “(D)(S/T)(N)” in TssB (see below). We were interested in determining if there was a preference for either of the two glycoforms, if there was a discernible pattern in glycoform distribution across the protein sequences, and if all available glycosylation sites were decorated with either of the two glycoforms or if some were prone to being left unglycosylated. To address these questions, a sample was prepared from extracted *T. serpentiformis* cells and pre-purified on a reversed-phase SPE tC18 cartridge, followed directly by LC–electrospray ionization (ESI)–MS/MS. This sample represented a baseline state that would reveal any occupied as well as unoccupied glycosylation sites. In both TssA and TssB, most detected glycosylation sites were indeed occupied by one of the two glycoforms, and only a few sites were mainly left unglycosylated (1 out of 14 detected and verified sites for TssA and none out of 21 detected and verified sites for TssB was primarily unglycosylated). For TssA, five sites were mainly glycosylated and only occasionally unglycosylated (Supplementary Fig. S19). Some sites in TssA appeared to a lesser degree in an unglycosylated state (five sites with under 20% detection), but none in TssB. Interestingly, the one unglycosylated site in TssA was the most N-terminally detected site and the second most N-terminally predicted site. However, the most N-terminally predicted glycosylation site in TssA at position 30 (“DSV”) was not reliably detected in MS, likely due to cleavage of part of the N-terminal amino acid sequence as a signal peptide during protein export. In TssB, the detected glycosylation site closest to the N-terminus was at position 61 (“DSV”). The predicted signal peptides for TssA and TssB, as predicted by DeepTMHMM (Hallgren et al. 2022), are MNKKVFTLLAASFMLLLGAVGASA (first 24 amino acids) and MIMNKKIFTLLAGILMLGLFAVSGNA (first 26 amino acids), respectively. The most N-terminal verified fragment in the MS data started at residue 58 for TssA and at residue 54 for TssB. Additionally, the C-terminal region in both proteins offered no potential glycosylation sites. Notably, in both S-layer proteins, the occupied and detected glycosylation sites closest to the C-terminus were more than 150 residues away from the C-terminus (*i.e*., 195 amino acids for TssA and 176 amino acids for TssB).

Among all detected and occupied sites in TssA and TssB, there was typically a strong preference for one of the two glycoforms (or an unglycosylated state) instead of an even glycosylation distribution, except for one case each in TssA and TssB, where the distribution was shared between both glycoforms (3:1 in TssA and 1:1 in TssB, G1:G2, respectively).

Interestingly, during analysis of glycosylation site occupancy in TssB, we discovered a clear indication of glycosylation on peptides carrying a “D/(S/T)/N” motif, which was not yet part of the amino acid motif at the time of analysis. In total, four instances of this motif could be verified in TssB, two of DSN and DTN, each. This motif is not present in TssA.

Regarding the spatial distribution of occupied glycosylation sites along the linear amino acid sequence, there is a noticeable decline of available (and occupied) glycosylation sites in both proteins towards the C-terminus, with the last occupied sites in TssA at position 995 (out of 1,190 residues) and in TssB at position 1,220 (out of 1,396 residues). The two available glycoforms are distributed among the occupied sites with no discernable pattern. No glycoform is particularly enriched in any region of the amino acid sequence (Supplementary Fig. S19).

**Fig. 6:**
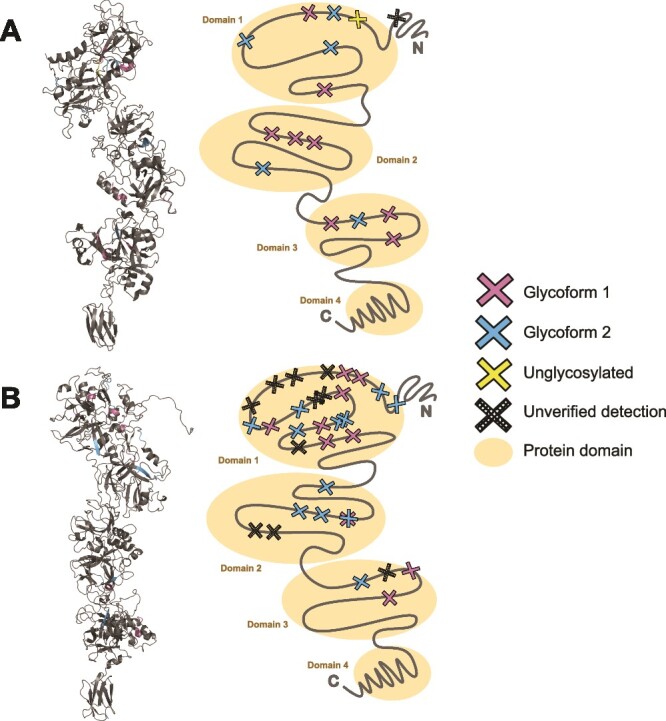
3D-distribution of glycoform G1 and G2 on the Alphafold2-generated structures of the S-layer proteins. **A**) TssA (feature BCB71_RS00675 in GenBank: CP017038.2, UniProt A0A2R4KII9) and **B**) TssB (feature BCB71_RS00680 in GenBank: CP017038.2, UniProt A0A2R4KIF3) as determined by LC-MS/MS. Potential glycosylation sites where spectra did not pass manual validation were marked as “unverified detection.” Glycoforms are distributed equally across the first (most N-terminal), second, and third domain, where the majority of available sites resides in the first and largest domain. The fourth and most C-terminal domain is left unglycosylated. Left, protein structures are shown as “cartoon” representation. Structures visualized with pyMOL (Schrödinger and DeLano 2020).

To investigate the spatial glycosylation site distribution in 3D space, an Alphafold2 model was generated for each TssA and TssB based on the published protein sequences (Frey et al. 2018) and the translated genomic features (BCB71_RS00675 for TssA and BCB71_RS00680 for TssB), which are identical to the subsequently identified protein sequences listed in the UniProt entries A0A2R4KII9 and A0A2R4KIF3, respectively (Fig. 6A and B). The occupied sites are mainly located on the most N-terminal and largest domain in both cases. The most C-terminal and smallest of the four modelled S-layer protein domains, which is cleaved in the course of protein secretion *via* the type IX protein secretion system (T9SS) based on its typical structural features (Tomek et al. 2014), is completely devoid of any available glycosylation sites. The two domains in between are moderately glycosylated in either case, mainly because fewer sites are available for glycosylation. Overall, just like with the distribution of glycosylation sites along the linear amino acid sequence, there was no discernible pattern of distribution between the two glycoforms based on predicted protein folding.

## Discussion

**Fig. 7:**
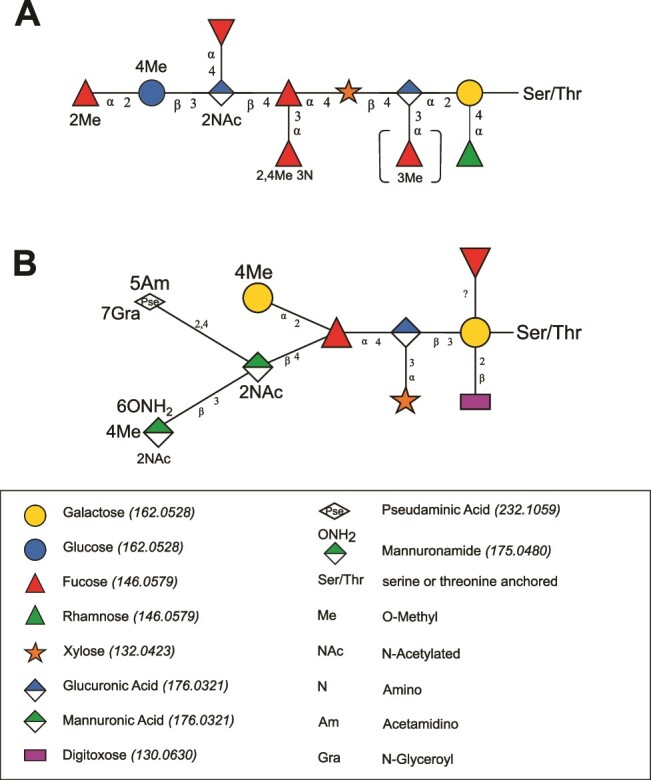
Comparison of the major *O*-glycan structures from **A**) *T. serpentiformis* (this study) and **B**) *T. forsythia* (Posch et al. 2011; Tomek et al. 2018); both are linked to serine or threonine residues within an extended *Bacteroidetes* glycosylation motif (this study and (Ye et al. 2024)). Most notable differences lie in absence of nonulosonic acid, the high degree of fucosylation, and the frequency of modifications in the *T. serpentiformis O*-glycan. The structures were generated with GlycanBuilder2 (Tsuchiya et al. 2017). Monosaccharide symbols adhere to the SNFG (Varki et al. 2015). Numbers in parenthesis refer to underivatized monoisotopic masses in Dalton.

The *O*-glycan structure of *T. serpentiformis* was fully elucidated using NMR spectroscopy, revealing notable distinctions from its counterpart in pathogenic *T. forsythia* (Tomek et al. 2018) in terms of composition and structure (Fig. 7). This divergence is reflected by differences in the genomic protein glycosylation gene clusters (Zwickl et al. 2020). Moreover, our comprehensive glycoproteomic analysis within this study reaffirmed the established predicted *Bacteroidetes* phylum-wide three-amino acid *O*-glycosylation motif at serine and threonine sites, identified as D(S/T)(L/V/T/A/I) in *Tannerella* species. Importantly, our acquired MS data revealed a novel candidate “N” occupying the third position of the motif (Supplementary Fig. S19).

In *T. serpentiformis*, we identified two major, branched glycoforms- a decasaccharide (G1) and an undecasaccharide (G2), which differ by one *O*-methylated fucose (Fig. 3, Supplementary Fig. S5). Each glycoform comprises seven different monosaccharide types, many of which carry non-carbohydrate modifications, predominantly methyl groups. Notably, one particular fucose exhibits a rare modification, featuring methoxy-groups at the C2 and C4 and an amino group at the C3. This sugar modification has only been reported once before in the literature, specifically in a cell wall antigen of the Gram-positive oral bacterium *Eubacterium saburreum*, albeit without indication of functional implications (Jansson et al. 1985). Overall, the *T. serpentiformis O*-glycan (this study) is a complex oligosaccharide, akin to that of *T. forsythia* (Posch et al. 2011; Tomek et al. 2018) (Fig. 7), which has been shown to exist in multiple glycoforms (Veith et al. 2021). This macro- and microheterogeneity is intrinsic to the concept of bacterial protein glycosylation. The absence of nonulosonic acid in the *T. serpentiformis* glycoforms aligns with previous findings suggesting that this sugar acid underpins the pathogenicity potential of *T. forsythia*, *e.g.*, by evading immune recognition (Settem et al. 2013; Tomek et al. 2018) or aiding the establishment in the oral biofilm consortium (Bloch et al. 2017); *vice versa*, its absence supports the health-associated properties of *T. serpentiformis* (Frey et al. 2018; Ansbro et al. 2020; Kendlbacher et al. 2023).

Remarkably, all structurally elucidated (*via* NMR spectroscopy) or predicted (supported by MS analysis) *O*-glycans from members of the *Bacteroidetes* phylum, including *Elizabethkingia meningoseptica* (*Flavobacterium meningosepticum*) (Reinhold et al. 1995), *Flavobacterium columnare* (Vinogradov et al. 2003), *Bacteroides fragilis* (Tomek et al. 2021), *P. gingivalis* (Veith et al. 2022), *P. intermedia* (Ye et al. 2024), and *T. forsythia* (Posch et al. 2011; Tomek et al. 2021; Veith et al. 2021), share a common feature in their structural arrangement at the reducing end, a protein-linked hexose (either galactose or mannose in elucidated structures) carrying a branching deoxy-hexose (fucose or rhamnose in elucidated structures) and a proximal uronic acid (glucuronic acid). This structural detail might be regarded as a core saccharide (Coyne et al. 2013), suggesting possible biosynthetic implications. Notably, a step-wise glycan truncation approach demonstrated for *T. forsythia* that no biosynthetic enzymes for this “core” are encoded within the *O-*glycosylation gene cluster (Tomek et al. 2018).

A notable characteristic of the *T. serpentiformis O*-glycan is its extensive fucosylation and its presence on cell surface associated proteins (Fig. 7A, Supplementary Figs S1 and S5). Terminal fucosylation is employed by various pathogens to integrate into the host environment and escape immediate detection by the host immune system (Thomès and Bojar 2021). For instance, *B. fragilis* decorates numerous capsular polysaccharides and glycoproteins with l-fucose to gain a competitive advantage in colonization (Coyne et al. 2005). In the *T. serpentiformis O-*glycan, four out of five fucoses are accessible; three fucoses, including the terminal fucose, undergo multiple modifications (Fig. 7A). It is reasonable to assume that these fucose residues are l-fucoses, given the presence of both biosynthesis genes for l-fucose in the *T. serpentiformis* genome-*gmd* encoding GDP-mannose 4,6-dehydratase (UniProt A0A2R4KF50 and locus tag BCB71_06850) and *fcl* encoding GDP-l-fucose synthase (UniProt A0A2R4KF49 and locus tag C7123_01625). The modifications of the fucose residues may mitigate the mimicking effect achieved by extensive fucosylation. The absence of carbohydrate mimicry might partly explain the increased production of various pro-inflammatory mediators by host cells when exposed to *T. serpentiformis* (Kendlbacher et al. 2023). It is worth noting that the development and utilization of highly specific fucosylated glycans through modifications, such as methylation, are considered as strategies of distinct organisms to recognize their preferred interaction partners or to attract species with highly specific recognition systems (Thomès and Bojar 2021). *T. serpentiformis* might employ this strategy when integrated in the oral biofilm consortium, where it has been observed to co-exist with some biofilm bacteria while outcompeting others, including pathogenic species like *T. forsythia* and *P. gingivalis* (Kendlbacher et al. 2023). However, the precise role of fucosylation in this process requires further investigation.

When considering the precise positioning of either of the two glycoforms on the two *T. serpentiformis* S-layer proteins TssA and TssB (Fig. 6, Supplementary Fig. S16), no clear pattern emerges. Prokaryotic S-layer glycosylation may serve various purposes, including cell–cell signaling, host immune response modulation, structural S-layer stabilization, or formation of biofilms (Bharat et al. 2021). Therefore, further investigation is necessary before the specific rationale behind glycosylation events at particular sites, or the absence thereof, can be fully understood.

Regarding the macro-heterogeneity of glycan distribution, glycosylation site ccupancy tended to be one-sided, with sites either glycosylated with a preferred glycoform or remaining unglycosylated. An equal distribution of glycosylation state was rarely observed. It should be noted that the presence and location glycosylation (C-terminal, N-terminal, or in the middle) on a peptide can influence ionization strength compared to the same unglycosylated peptide (Stavenhagen et al. 2013). Signal strengths of glycopeptides are often reduced, especially for complex glycans, compared to their unglycosylated counterparts. Therefore, in this study, the relative amount of “G1 peptide” or “G2 peptide” may be underestimated compared to the “unglycosylated” portion. Determining the exact quantitative ratios of glycosylated and unglycosylated peptides was beyond the scope of this study, particularly given the focus on the complex *O*-linked glycans. Thus, the ratios of G1 peptide:G2 peptide:unglycosylated peptide should be considered indicative rather than exact representations. Nevertheless, the general tendencies regarding macro-heterogeneity remain consistent.

In terms of amino acid glycosylation motif, we have confirmed that a foundational motif of (D)(S/T)(L/A/V/T/I) can be reliably established for members of the *Bacteroidetes* phylum and especially *Tannerella* species (Fig. 5, Supplementary Figs S16–S18). This fundamental motif was defined through a meticulous selection process involving a high-density dataset and objective criteria. The only externally defined criterion was the second position (S/T), recognized as the typical *O-*glycosylated amino acids in prokaryotes, although we acknowledge that tyrosine is a viable albeit rare alternative (Zarschler et al. 2010). Utilizing probability theory, specifically through the IceLogo tool, adds an additional layer of unbiasedness comparing significantly overrepresented amino acids in a three-letter motif to an unglycosylated reference proteome, thus eliminating any inherent amino acid preferences within the organism. Furthermore, in our experimental approach, *T. serpentiformis* glycopeptide fragments were enriched using an HILIC column rather than filtering a dataset based on an externally predefined amino acid motif. Therefore, the motif (D)(S/T)(L/A/V/T/I) establishes a common framework for *T. serpentiformis* and *T. forsythia,* rather than for all members of the *Bacteroidetes* phylum, and it does not exhaustively list all potential amino acid options in each of the three positions. Additionally, this study introduces “N” as an additional candidate for the third and most variable position in the glycosylation amino acid motif. We emphasize that the data generated in this study, based on the two closely related species *T. serpentiformis* and *T. forsythia,* reflect a clear preference for specific amino acids in the third position. While other amino acid candidates may be present in rare cases, they do not significantly contribute to the preferred glycosylation amino acid motif. Thus, it is important to note that other members of the *Bacteroidetes* phylum may exhibit a broader selection of amino acid candidates for the third position in the glycosylation amino acid motif, influenced by the organism’s amino acid preference and glycosylation machinery.

In summary, this study elucidated the glycan structure of the two main glycoforms in *T. serpentiformis* and confirmed the amino acid motif (D)(S/T)(L/V/T/A/I) as a reliable baseline motif in the *Bacteroidetes* phylum, and especially in *T. serpentiformis* and *T. forsythia.* Furthermore, based on the two S-layer proteins in *T. serpentiformis*, the amino acid “N” was established as a candidate for the third position, a novel finding at the time that has since been corroborated by colleagues in another member of the phylum (Ye et al. 2024), summing up to a total of 15 candidates for the third position (Ye et al. 2024). Notably, this number corresponds to effectively 75% of all main amino acids, leading the authors to question the existence or significance of a “phylum-wide” three-amino acid glycosylation motif. Instead, the data presented in this study indicate a strong preference for a small number of amino acids within one species and its closest phylogenetic relative, suggesting that three-amino acid glycosylation motives might be more genus-specific than universal. Consequently, the most broadly applicable motif within the *Bacteroidetes* phylum might be a two amino-acid one: X-D-S/T-X.

The distribution of the two glycoforms on the two S-layer proteins was closely investigated. However, no discernable distribution pattern based on the linear amino acid sequence or 3D representation of each protein has been identified.

## Conclusion

The complex *O*-glycan structure and composition elucidated in this study underscore the health-associated status that *T. serpentiformis* holds in the oral biofilm community. Data specific to *T. serpentiformis* enabled us to confirm the recently identified candidate “N” for the glycosylation amino acid motif in the *Bacteroidetes* phylum, while also establishing a baseline motif that is likely shared between several members of the phylum. It is noteworthy that the complexity of the amino acid motif, particularly concerning the third position, continues to increase, suggesting that the selection of occupied sites may be influenced by organism-specific biases. Finally, further investigations into the logic of glycosylation events may illuminate the role of S-layer glycosylation more broadly.

## Materials and methods

### Bacterial cultivation conditions

*Tannerella serpentiformis* W11667 (provided by Dr. Graham Stafford, Integrated BioSciences, School of Clinical Dentistry, University of Sheffield, UK) was grown anaerobically on Fastidious Anaerobe agar (FAA; Neogen), supplemented with 5% horse blood (Sigma-Aldrich) and 20 μg/mL *N*-acetylmuramic acid (NAMA; Sigma-Aldrich) (Vartoukian et al. 2016) for 6-7 days at 37 °C. Bacteria were harvested by scraping them off the plates, followed by washing the pellet in 1x phosphate-buffered saline (PBS, Sigma-Aldrich). Bacterial pellets were stored at −20 °C until further processing.

### Glycoprotein extraction

Proteins were extracted from bacterial cells using the chloroform-methanol method (Wessel and Flügge 1984). Approximately 2.5 g of bacteria (wet weight) were suspended in 4-(2-hydroxyethyl)-1-piperazineethanesulfonic acid (HEPES) buffer (100 mM, pH 7.8, Sigma-Aldrich) and vortexed with chloroform (Roth), methanol (Honeywell | Riedel-de Haën) and deionized water (1:6:3, v/v/v). The suspension was then centrifuged for 10 min at 15,000 × g in a fixed-angle tabletop centrifuge (Eppendorf 5427 R) to achieve phase separation. The extracted protein pellet was air-dried for 1.5 h before being resuspended in absolute ethanol (AustrAlco Österreichische Agrar-Alkohol Handelsges.m.b.H.); subsequently, the suspension was evaporated in a rotavapor (Büchi) to remove any residual chloroform from the sample.

### Glycopeptide preparation

The extracted proteins were dissolved in Pronase E buffer (0.15 M Tris/HCl (Trizma Base; Sigma-Aldrich) pH 7.6, 1 mM CaCl_2_ (Roth)) (2% w/v) and incubated at 80 °C in a water bath for 30 min. Afterwards, the solution was cooled down on ice before adding Pronase E from *Streptomyces griseus* (Fluka) in Pronase E buffer at a ratio of 1:50 (w/w, enzyme:sample). Half of the required enzyme amount was initially added to the sample, followed by incubation at 37 °C for 2 h. Subsequently, the remaining enzyme was added, and the digestion was allowed to proceed overnight at 37 °C. The mixture was then reduced to approximately 1 mL using a rotavapor (Büchi) for further processing.

### Glycopeptide purification

Glycopeptides were initially pre-purified on a coarse-grain Sephadex G-25 gel filtration column (50 mL; 2.6 cm in diameter; running buffer: 1% acetic acid, flow rate: 1 mL/min, GE Healthcare). Fractions of 1 mL we collected, and aliquots were spotted on TLC silica aluminum plates (Macherey-Nagel) and stained with orcin for carbohydrates (0.2% orcinol monohydrate in 10% sulfuric acid in absolute ethanol). The protein concentration was estimated using UV spectroscopy at 280 nm and 235 nm in clear 96-well flat bottom cyclic olefin copolymer (COC) plates (Tecan infinite 200 Proreader, Greiner plates). Combined carbohydrate-positive fractions were then reduced to about 1 mL in a rotavapor (Büchi) and checked for successful digestion by SDS-PAGE using a 15% polyacrylamide gel (Laemmli 1970) in a Miniprotean apparatus (Biorad), run at 180 V for 1 h, followed by protein staining with Coomassie Brilliant Blue G250 (CBB) and destaining in 10% acetic acid overnight. Subsequently, samples were further purified using methanol-primed and conditioned reversed-phase solid-phase cartridges (SPE tC18 RP, Waters, 200 mg) with 0.1% trifluoro acetic acid (TFA). The flowthrough along with a 1-mL washing fraction with deionized water were collected and combined for subsequent purification on an RP cartridge (Superclean ENVI-Carb SPE Tube, Sigma-Aldrich). Samples were eluted in 50% aqueous acetonitrile (Optima LC/MS grade, Fisher Scientific) and, for control by MALDI-TOF MS, in 50% acetonitrile in 0.1 M aqueous NaHCO_3_. The (glyco)peptides were dried and re-suspended in 50 μL 80% acetonitrile containing 1% TFA, and fractionated using a TSK-Amide 80 column (4.6 × 250 mm, 5 μ, Tosoh) with a linear gradient from 80% acetonitrile containing 0.1% TFA to 50% acetonitrile containing 0.1% TFA at a flow rate of 1 mL/min (fraction size, 1 mL) over 30 min. Fractions were spotted on TLC silica aluminum sheets, and orcin-positive fractions were combined and dried in a SpeedVac concentrator (Thermo-Scientific).

### β-Elimination of glycans

Glycans were released from purified glycopeptides by reductive β-elimination, employing 1 M sodium borohydride in 0.5 M sodium hydroxide overnight at 60 °C (Taylor et al. 2006; Posch et al. 2011). The β-eliminated glycans were subsequently purified on an RP cartridge (Superclean ENVI-Carb SPE Tube, Sigma-Aldrich) and eluted in 60% aqueous acetonitrile (Optima LC/MS grade, Fisher Scientific). The dried sample was then dissolved in D_2_O and analyzed by NMR spectroscopy.

### NMR spectroscopy of glycopeptides and β-eliminated glycans

The NMR spectra were acquired on a Bruker AV III HD 700 MHz NMR spectrometer (Bruker BioSpin, Ettlingen, Germany), equipped with a quadruple (^1^H, ^13^C, ^15^N, ^19^F) inverse helium cooled cryo probe operating at 700.40 MHz for ^1^H and 176.12 MHz for ^13^C, respectively.

All spectra were recorded at a temperature of 25 °C in D_2_O as solvent and referenced for ^1^H to the signal of the methyl groups of DSS (δ = 0 ppm). Chemical shifts for ^13^C are reported on a unified scale relative to ^1^H using the Ξ value for DSS (Harris et al. 2008). For all 1D and 2D NMR experiments, appropriate pulse sequences provided by the manufacturer were employed. ^1^H NMR spectra were acquired with suppression of the residual solvent signal using presaturation, while ^13^C NMR spectra were obtained using DEPTq sequence with a 135°-pulse for multiplicity selection. Subsequently, several 2D experiments were conducted, including double quantum filtered (DQF) COSY, TOCSY (100-ms MLEV17 spin-lock), NOESY (400-ms mixing time), HSQC with and without ^13^C decoupling, and HMBC. For the sequential assignment within the saccharides, the DQF-COSY method avoids dispersive contributions, providing the highest possible resolution with undistorted fine structure, even for cross-peaks near the diagonal. To obtain high-resolution spectra of the individual sugar spin systems, frequency selective experiments were performed. In conventional 1D TOCSY, a selective pulsed field gradient spin-echo sequence with an 80-ms 180° Gaussian pulse for excitation of an uncovered signal was utilized, followed by a MLEV17 spin-lock with a duration between 100 and 300 ms, depending on the size of the spin–spin coupling. In cases where all signals of a sugar of interest were overlapped, the DREAMTIME selection scheme was applied (Jenne et al. 2022). This involved using a double selective 40-ms 180° Gaussian pulse on the frequencies of two *J*-coupled spins simultaneously, followed by a double quantum filter to extract only the desired spin pairs. The resulting in-phase magnetization can be directly recorded, providing a spectrum of the two coupled sugar protons, or subjected to a TOCSY spin-lock (DIPSI, 100 to 300 ms) to generate the entire spin system. The analysis of the ^1^H spin couplings was supported by spin simulations using the TopSpin software (Bruker Biospin, Ettlingen, Germany), fitting the calculated spectra to the experimental 1D TOCSY traces.

### Glycoproteomics sample preparation

Glycoproteomic analysis was performed on both extracted *T. serpentiformis* cells and S-layer glycoprotein bands excised from SDS PAGE gels.

Briefly, *T. serpentiformis* cell pellets (10 mg) were resuspended in 80 μL 1× Laemmli buffer (125 mM Tris/HCl, 4% SDS, 2% β-mercaptoethanol, 20% glycerin, pH 6.8), boiled for 5 min in a water bath, and 4 μL (~0.5 mg) (glyco)proteins were loaded per well and separated on a 7.5% SDS-PAGE (Laemmli 1970). Following CBB-staining, two high-molecular-mass S-layer glycoprotein bands were excised, minced, de-stained, and dried in a SpeedVac concentrator. Next, gel pieces were subjected to carbamidomethylation in 10 mM DL-dithiothreitol (DTT, Sigma-Aldrich) in 100 mM aqueous ammonium bicarbonate buffer for 45 min at 56 °C, with shaking at 1,000 rpm. Subsequently, 55 mM iodacetamide (Sigma-Aldrich) in 100 mM ammonium biocarbonate buffer was added, and the mixture was incubated for 1 h in the dark. After washing, the gel pieces were tryptically digested overnight at 37 °C (150 ng protease in 25 mM ammonium bicarbonate buffer per sample; sequencing-grade trypsin, Promega) (Posch et al. 2011). Peptides were then extracted sequentially by addition of 25 mM ammonium bicarbonate buffer, 100% acetonitrile (Optima LC/MS grade, Fisher Scientific), and 5% formic acid. The resulting supernatant containing the tryptic (glyco)peptides was evaporated in a SpeedVac concentrator for subsequent MS analysis.

After conducting glycoproteomic analysis of the excised gel bands and aligning identified fragments with UniProt IDs (see Data Analysis section below), it was discovered that a plethora of glycoproteins, including but not limited to the suspected S-layer proteins, were present in the target bands. Consequently, the entire proteome of *T. serpentiformis* was analyzed following the extraction of (glyco)proteins from a bacterial cell pellet (20 mg, wet weight) by chloroform-methanol as described above (Wessel and Flügge 1984). The resulting dried samples (SpeedVac concentrator) were then reconstituted in 4-(2-hydroxyethyl)-1-piperazineethanesulfonic acid (HEPES) buffer (100 mM, pH 7.8, Sigma-Aldrich) for digestion with sequencing-grade trypsin (Sigma-Aldrich). The sample was reduced in 25 mM freshly prepared DL-dithiothreitol (DTT, Sigma-Aldrich) and incubated at 54 °C with shaking at 2,000 rpm in a heat block for 30 min. Subsequently, 25 mM iodoacetamide in deionized water was added, and the sample was incubated in the dark for 1 h. Sequencing-grade trypsin (Sigma-Aldrich) was then added at a ratio of 1:50 to 1:100 (enzyme:sample, w/w) and sample was further incubated overnight at 37 °C. The resulting tryptic (glyco)peptides were isolated using a methanol-primed and conditioned (0.1% TFA) C18 reversed-phase SPE cartridges (Waters, 200 mg) and eluted from the cartridge in 50% acetonitrile (Optima LC/MS grade, Fisher Scientific) in 0.1% aqueous formic acid. The dried, C18-SPE-purified glycopeptides were subsequently enriched as described above for NMR glycopeptide samples. IP-HILIC fractions 25–39 were collected and dried in a Speed-Vac concentrator.

### LC-ESI-MS/MS analysis of glycopeptides

The dried IP-HILIC fractions were resuspended in 20 μL of 0.1% formic acid and subjected to RP separation using a nanoEase M/Z HSS T3 column (100 Å, 1.8 μm, 300 μm × 150 mm; Waters). The separation was achieved by employing a linear gradient from 0.8% acetonitrile in 0.1% formic acid to 32% acetonitrile and 0.1% formic acid over 80 min at a flow-rate of 6 μL/min, followed by a 5-min linear gradient from 32% acetonitrile and 0.1% formic acid to 76% acetonitrile and 0.1% formic acid. Data-dependent MS/MS analysis was conducted using an Orbitrap Exploris 480 (Thermo) instrument, equipped with its standard H-ESI source operated in positive mode. The instrument was configured with the following settings: MS data was acquired in the mass-range from 350 to 1,500 at a resolution of 60,000; MS/MS data (isolation window 1.4 mu; isolation offset 0.5 mu, normalized AGC target 200%) were automatically acquired for (glyco)peptide precursor ions of charge-states ranging from 2 up to 6 and above a threshold of 80,0000, using stepped HCD (normalized collision energies of 28, 30, and 35) in centroid mode, with the first fixed mass at *m/z* 120, at a resolution of 30,000.

### Data-analysis and glycopeptide identification

Raw MS/MS data were extracted, refined (including precursor mass and charge-state; no scan merging), and converted into the .mgf file-format using PEAKS X Pro Studio 10.6 (build 20201221). Subsequently, custom-coded perl-scripts (“SugarQBits”; all scripts are freely available at http://homepage.boku.ac.at/jstadlmann) were utilized for glycopeptide-specific “open-search” analysis. In essence, all charge-deconvoluted and deisotoped MS/MS data were scrutinized for the co-occurrence and intensities of three diagnostic fragment ion signals (mass-precision ±10 ppm), which potentially corresponded to the prominent glycopeptide Y2-fragment ion (*i.e*., [peptide+Hex+HexA+H]^+^ and the neutral losses of 338.0849 amu and 176.0321 from the respective Y2-ion candidate). Of MS/MS spectra containing all three fragment ion signals, the precursor MS/MS information (stored in .mgf file-format as PEPMASS) was adjusted to the mass of the putative Y1-fragment ion detected using “SugarQBits_Kassonade.pl”; subsequently, a series of empirically determined glycan-specific oxonium fragment ion signals (i.e. 126.0547, 142.0861, 157.0857, 174.1122, 200.0552, 218.0656 amu) were removed using “SugarQBits_RepX” (mass-precision ±10 ppm). The amino-acid sequences of glycopeptides were identified from the pre-processed .mgf-files using Comet, implemented in SearchGUI (version: 4.1.11) (Barsnes and Vaudel 2018), employing a species-specific proteome sequence data-base (*i.e.*, *T. serpentiformis* UP000240373; uniprot.org; 2,359 proteins), concatenated with their respective reversed sequences as decoys. The search engine settings included semi-tryptic digest, allowing up to 1 missed cleavage, carbamidomethylation as a fixed modification of all cysteines, oxidation as variable modification to all methionine residues, and [Hex+HexA] (including the neutral loss of 338.0849 amu) as a variable modification to all serine and threonine residues. Spectral matching was performed with a mass-precision of ±10 ppm on the precursor level, and ±0.05 amu at the MS/MS level. Contaminants were not considered or excluded. Glycopeptide identifications (search-engine rank 1, peptide length greater than 7, and at least one [Hex+HexA]-modified residue) were manually filtered to an approximately 1% false-discovery-rate at the spectrum level, using the target-decoy approach (Elias and Gygi 2007), as reported previously (Stadlmann et al. 2017). No site localization of glycans was performed.

Glycan-mass histograms were constructed from the determined mass differences between the original glycopeptide precursors and the identified peptide-sequences of all glyco-PSMs (FDR < 1%), at a bin-width of 0.1 amu, using spectral counting automatically (SugarQBits_Kassonade). For glycosylation-site specific glycan-mass histograms, only glyco-PSMs (FDR < 1%) covering the respective site were counted.

### Glycosylation site occupancy

To assess the occupancy of glycosylation sites, a reference sample was prepared identically to the whole-cell preparation for glycoproteomic samples (described above), excluding the final glycopeptide enrichment step via HILIC. This sample represented a “baseline” dataset encompassing all (glyco)peptides extracted from *T. serpentiformis* cells.

Each identified peptide fragment (as described in the section “LC-ESI-MS/MS analysis of glycopeptides”) from the two S-layer proteins, TssA (feature BCB71_RS00675 in GenBank: CP017038.2, UniProt A0A2R4KII9) and TssB (feature BCB71_RS00680 in GenBank: CP017038.2, UniProt A0A2R4KIF3), underwent manual validation using the Skyline software (MacLean et al. 2010). The normalized areas for each glycopeptide entry were compared to establish a profile of glycoform occupancy. Three peptide states were reported: unglycosylated (peptide only), glycoform 1 (+1634.6 variable modification) and glycoform 2 (+1794.6 variable modification).

### Glycosylation motif analysis

For the *in-silico* analysis of (occupied) glycosylation amino acid motifs, a mass spectrometry dataset derived from bacterial whole cell preparations was used, excluding duplicated sequences.

Common amino acid triplets among the identified glycopeptide sequences were determined using IceLogo (Colaert et al. 2009), a probability-based analysis tool for visualizing consensus sequences in multiple aligned peptide sequences. The application compares a given sample set (*e.g*., a list of three-letter amino acid groups) to a reference set (*e.g*., a reference proteome containing only unglycosylated proteins) and identifies over- and under-represented amino acids to generate a statistically significant motif (*P* = 0.05 for all analyses). In this study, the background (“reference”) set of sequences was created by downloading the full *T. serpentiformis* (W11666) proteome from the UniProt database (UP000240373), comprising 2,360 entries. Subsequently, all entries associated with glycoproteins identified by MS analysis in this study were removed, resulting in a dataset representing evidently unglycosylated protein sequences in *T. serpentiformis* (“delta population”).

The sample set utilized in this study comprised all identified glycopeptide sequences, which were also present in the complete proteome dataset available at UniProt. This dataset was compiled in one of two ways. Firstly, all sequence triplets with either “S” or “T” (serine or threonine, typical for *O-*glycosylation) in the second position were collected, leaving positions 1 and 3 of the motif to be investigated ((X)(S/T)(X)). Motifs were searched within glycoprotein fragments identified in this experiment, rather than within full glycoprotein sequences, to construct the sample dataset. This approach was chosen due to statistical constraints in comparison to the reference dataset. Secondly, to analyze the complete motif within the full glycoprotein sequences, a sample set of three amino acids containing “D” in position 1 and “S” or “T” in position 2 was generated [(D)(S/T)(X)]. Here, motifs were searched in the identified glycopeptide fragments as well as in the full glycoprotein sequences associated with identified UniProt IDs, as the sample dataset size was adequate compared to the reference dataset size.

This approach was validated by performing the same steps on a publicly available dataset for *T. forsythia* (Veith et al. 2021), where similar glycoproteomics data were used to establish a putative phylum-wide glycosylation motif. A reference dataset containing the complete *T. forsythia* proteome publicly available on UniProt (taxon 28112, UP000005436), comprising 2,979 entries, was created, excluding any entries containing UniProt IDs associated with glycoproteins identified by Veith *et al.* in their study. This process generated an “unglycosylated delta-proteome” as a background dataset. The sample dataset consisted of either “(X)(S/T)(X)” or “(D)(S/T)(X)” amino acid triplets derived from the glycopeptide fractions provided by the authors in their Supplementary material or the full glycoprotein sequences derived from the *T. forsythia* proteome as described above, depending on the positions to be queried in the motif.

### Protein structure prediction

Models for TssA (feature BCB71_RS00675 in GenBank: CP017038.2, UniProt A0A2R4KII9) and TssB (feature BCB71_RS00680 in GenBank: CP017038.2, UniProt A0A2R4KIF3) were predicted using an in-house installation of Alphafold version 2.0.1, employing default settings for monomers. Five models were generated with relaxation enabled. Model 0, ranked highest, was selected for further analysis for TssA, while for TssB, due to memory constraints, relaxed model 1 was chosen as no ranked model could be generated. Overall, the predictions for both models were deemed acceptable, with average pLDDT’s of 81.49 for TssA and 74.35 for TssB.

## Supplementary Material

SI_GLYCO-2024-00072_13092024_cwae072

## Data Availability

Data supporting the findings of this study are provided in the article and as Supplementary Material. Raw data of MS analysis is deposited in GlycoPOST (Watanabe et al. 2020), submission ID: GPST000434. URL: https://glycopost.glycosmos.org/preview/21059169826698ecfac4458, PIN CODE: 4998.

## References

[ref1] Aeschbacher T, Zierke M, Smieško M, Collot M, Mallet JM, Ernst B, Allain FH, Schubert M. A secondary structural element in a wide range of fucosylated glycoepitopes. Chemistry. 2017:23(48):11598–11610. 28654715 10.1002/chem.201701866

[ref2] Ansbro K, Wade WG, Stafford GP. *Tannerella serpentiformis* sp. nov., isolated from the human mouth. Int J Syst Evol Microbiol. 2020:70(6):3749–3754. 32519941 10.1099/ijsem.0.004229

[ref3] Barsnes H, Vaudel M. SearchGUI: a highly adaptable common interface for proteomics search and de novo engines. J Proteome Res. 2018:17(7):2552–2555. 29774740 10.1021/acs.jproteome.8b00175

[ref4] Beall CJ, Campbell AG, Griffen AL, Podar M, Leys EJ. Genomics of the uncultivated, periodontitis-associated bacterium *Tannerella* sp. BU045 (oral taxon 808). mSystems. 2018:3(3):e00018. 29896567 10.1128/mSystems.00018-18 PMC5989130

[ref5] Bharat TAM, von Kügelgen A, Alva V. Molecular logic of prokaryotic surface layer structures. Trends Microbiol. 2021:29(5):405–415. 33121898 10.1016/j.tim.2020.09.009 PMC8559796

[ref6] Bloch S, Thurnheer T, Murakami Y, Belibasakis GN, Schäffer C. Behavior of two *Tannerella forsythia* strains and their cell surface mutants in multispecies oral biofilms. Mol Oral Microbiol. 2017:32(5):404–418. 28382776 10.1111/omi.12182 PMC5600126

[ref7] Bloch S, Tomek MB, Friedrich V, Messner P, Schäffer C. Nonulosonic acids contribute to the pathogenicity of the oral bacterium *Tannerella forsythia*. Interface Focus. 2019:9(2):20180064. 30842870 10.1098/rsfs.2018.0064 PMC6388019

[ref8] Colaert N, Helsens K, Martens L, Vandekerckhove J, Gevaert K. Improved visualization of protein consensus sequences by iceLogo. Nat Methods. 2009:6(11):786–787. 19876014 10.1038/nmeth1109-786

[ref9] Coyne MJ, Reinap B, Lee MM, Comstock LE. Human symbionts use a host-like pathway for surface fucosylation. Science. 2005:307(5716):1778–1781. 15774760 10.1126/science.1106469

[ref10] Coyne MJ, Fletcher CM, Chatzidaki-Livanis M, Posch G, Schäffer C, Comstock LE. Phylum-wide general protein *O*-glycosylation system of the *Bacteroidetes*. Mol Microbiol. 2013:88(4):772–783. 23551589 10.1111/mmi.12220 PMC3656502

[ref11] Dominy SS, Lynch C, Ermini F, Benedyk M, Marczyk A, Konradi A, Nguyen M, Haditsch U, Raha D, Griffin C, et al. Porphyromonas gingivalis in Alzheimer's disease brains: evidence for disease causation and treatment with small-molecule inhibitors. Sci Adv. 2019:5(1):eaau3333. 30746447 10.1126/sciadv.aau3333 PMC6357742

[ref12] Ebersole JL, Graves CL, Gonzalez OA, Dawson D 3rd, Morford LA, Huja PE, Hartsfield JK Jr, Huja SS, Pandruvada S, Wallet SM. Aging, inflammation, immunity and periodontal disease. Periodontol. 2016:72(1):54–75. 10.1111/prd.12135 27501491

[ref13] Elias JE, Gygi SP. Target-decoy search strategy for increased confidence in large-scale protein identifications by mass spectrometry. Nat Methods. 2007:4(3):207–214. 17327847 10.1038/nmeth1019

[ref14] Fletcher CM, Coyne MJ, Villa OF, Chatzidaki-Livanis M, Comstock LE. A general *O-*glycosylation system important to the physiology of a major human intestinal symbiont *Bacteroides fragilis*. Cell. 2009:137(2):321–331. 19379697 10.1016/j.cell.2009.02.041 PMC2772059

[ref15] Frey AM, Ansbro K, Kamble NS, Pham TK, Stafford GP. Characterisation and pure culture of putative health-associated oral bacterium BU063 (*Tannerella* sp. HOT-286) reveals presence of a potentially novel glycosylated S-layer. FEMS Microbiol Lett. 2018:365(17, 17):fny180. 10.1093/femsle/fny180 30052903

[ref16] Friedrich V, Janesch B, Windwarder M, Maresch D, Braun ML, Megson ZA, Vinogradov E, Goneau MF, Sharma A, Altmann F, et al. *Tannerella forsythia* strains display different cell-surface nonulosonic acids: biosynthetic pathway characterization and first insight into biological implications. Glycobiology. 2017:27(4):342–357. 27986835 10.1093/glycob/cww129 PMC5378307

[ref17] Hajishengallis G . Immunomicrobial pathogenesis of periodontitis: keystones, pathobionts, and host response. Trends Immunol. 2014:35(1):3–11. 24269668 10.1016/j.it.2013.09.001 PMC3947349

[ref18] Hajishengallis G . Periodontitis: from microbial immune subversion to systemic inflammation. Nat Rev Immunol. 2015:15(1):30–44. 25534621 10.1038/nri3785 PMC4276050

[ref19] Hallgren J, Tsirigos KD, Pedersen MD, Armenteros JJA, Marcatili P, Nielsen H, Krogh A, Winther O. DeepTMHMM predicts alpha and beta transmembrane proteins using deep neural networks. bioRxiv. 2022:2022. 10.1101/2022.04.08.487609.

[ref20] Harris RK, Becker ED, Menezes SMC, Granger P, Hoffman RE, Zilm KW. Further conventions for NMR shielding and chemical shifts (IUPAC recommendations 2008). Pure App Chem. 2008:80(1):59–84.

[ref21] Jansson P-E, Lindberg B, Spellman M, Hofstad T, Skaug N. The cell-wall antigen from *Eubacterium saburreum* strain L 13, a new type of biopolymer. Carbohydr Res. 1985:137:197–203.

[ref22] Jenne A, Bermel W, Michal CA, Gruschke O, Soong R, Ghosh Biswas R, Bastawrous M, Simpson AJ. DREAMTIME NMR spectroscopy: targeted multi-compound selection with improved detection limits. Angew Chemie. 2022:134(19):e202110044. 10.1002/anie.202110044 35170183

[ref23] Kendlbacher FL, Bloch S, Hager-Mair FF, Bacher J, Janesch B, Thurnheer T, Andrukhov O, Schäffer C. Multispecies biofilm behavior and host interaction support the association of *Tannerella serpentiformis* with periodontal health. Mol Oral Microbiol. 2023:38(2):115–133. 35964247 10.1111/omi.12385 PMC10947601

[ref24] Kinane DF, Stathopoulou PG, Papapanou PN. Periodontal diseases. Nat Rev Dis Primers. 2017:3:17038. 28805207 10.1038/nrdp.2017.38

[ref25] Laemmli UK . Cleavage of structural proteins during the assembly of the head of bacteriophage T4. Nature. 1970:227(5259):680–685. 5432063 10.1038/227680a0

[ref26] Lamont RJ, Hajishengallis G. Polymicrobial synergy and dysbiosis in inflammatory disease. Trends Mol Med. 2015:21(3):172–183. 25498392 10.1016/j.molmed.2014.11.004 PMC4352384

[ref27] MacLean B, Tomazela DM, Shulman N, Chambers M, Finney GL, Frewen B, Kern R, Tabb DL, Liebler DC, MacCoss MJ. Skyline: an open source document editor for creating and analyzing targeted proteomics experiments. Bioinformatics. 2010:26(7):966–968. 20147306 10.1093/bioinformatics/btq054 PMC2844992

[ref28] Marsh PD, Zaura E. Dental biofilm: ecological interactions in health and disease. J Clin Periodontol. 2017:44(Suppl 18):S12–S22. 28266111 10.1111/jcpe.12679

[ref29] Naginyte M, Do T, Meade J, Devine DA, Marsh PD. Enrichment of periodontal pathogens from the biofilms of healthy adults. Sci Rep. 2019:9(1):5491. 30940882 10.1038/s41598-019-41882-y PMC6445289

[ref30] Onishi S, Honma K, Liang S, Stathopoulou P, Kinane D, Hajishengallis G, Sharma A. Toll-like receptor 2-mediated interleukin-8 expression in gingival epithelial cells by the *Tannerella forsythia* leucine-rich repeat protein BspA. Infect Immun. 2008:76(1):198–205. 17967853 10.1128/IAI.01139-07 PMC2223669

[ref31] Park D-G, Woo BH, Lee B-J, Yoon S, Cho Y, Kim Y-D, Park HR, Song JM. Serum levels of interleukin-6 and titers of antibodies against *Porphyromonas gingivalis* could be potential biomarkers for the diagnosis of oral squamous cell carcinoma. Int J Mol Sci. 2019:20(11):2749. 31167516 10.3390/ijms20112749 PMC6600294

[ref32] Posch G, Pabst M, Brecker L, Altmann F, Messner P, Schäffer C. Characterization and scope of S-layer protein *O*-glycosylation in *Tannerella forsythia*. J Biol Chem. 2011:286(44):38714–38724. 21911490 10.1074/jbc.M111.284893 PMC3207478

[ref33] Posch G, Sekot G, Friedrich V, Megson ZA, Koerdt A, Messner P, Schäffer C. Glycobiology aspects of the periodontal pathogen *Tannerella forsythia*. Biomol Ther. 2012:2(4):467–482. 10.3390/biom2040467 PMC4030854 24970146

[ref34] Reinhold BB, Hauer CR, Plummer TH, Reinhold VN. Detailed structural analysis of a novel, specific O-linked glycan from the prokaryote *Flavobacterium meningosepticum*. J Biol Chem. 1995:270(22):13197–13203. 7768917 10.1074/jbc.270.22.13197

[ref35] Schrödinger L, DeLano W. 2020. PyMOL available at: http://www.pymol.org/pymol.

[ref36] Sekot G, Posch G, Oh YJ, Zayni S, Mayer HF, Pum D, Messner P, Hinterdorfer P, Schäffer C. Analysis of the cell surface layer ultrastructure of the oral pathogen *Tannerella forsythia*. Arch Microbiol. 2012:194(6):525–539. 22273979 10.1007/s00203-012-0792-3 PMC3354324

[ref37] Settem RP, Honma K, Nakajima T, Phansopa C, Roy S, Stafford GP, Sharma A. A bacterial glycan core linked to surface (S)-layer proteins modulates host immunity through Th17 suppression. Mucosal Immunol. 2013:6(2):415–426. 22968422 10.1038/mi.2012.85 PMC4049606

[ref38] Sharma A, Sojar HT, Glurich I, Honma K, Kuramitsu HK, Genco RJ. Cloning, expression, and sequencing of a cell surface antigen containing a leucine-rich repeat motif from *Bacteroides forsythus* ATCC 43037. Infect Immun. 1998:66(12):5703–5710. 9826345 10.1128/iai.66.12.5703-5710.1998 PMC108721

[ref39] Stadlmann J, Taubenschmid J, Wenzel D, Gattinger A, Dürnberger G, Dusberger F, Elling U, Mach L, Mechtler K, Penninger JM. Comparative glycoproteomics of stem cells identifies new players in ricin toxicity. Nature. 2017:549(7673):538–542. 28959962 10.1038/nature24015 PMC6003595

[ref40] Stafford G, Roy S, Honma K, Sharma A. Sialic acid, periodontal pathogens and *Tannerella forsythia*: stick around and enjoy the feast! Mol Oral Microbiol. 2012:27(1):11–22. 22230462 10.1111/j.2041-1014.2011.00630.x PMC4049603

[ref41] Stavenhagen K, Hinneburg H, Thaysen-Andersen M, Hartmann L, Silva DV, Fuchser J, Kaspar S, Rapp E, Seeberger PH, Kolarich D. Quantitative mapping of glycoprotein micro-heterogeneity and macro-heterogeneity: an evaluation of mass spectrometry signal strengths using synthetic peptides and glycopeptides. J Mass Spectr. 2013:48(6):627–639. 10.1002/jms.3189 23776102

[ref42] Taylor AM, Holst O, Thomas-Oates J. Mass spectrometric profiling of *O*-linked glycans released directly from glycoproteins in gels using in-gel reductive beta-elimination. Proteomics. 2006:6(10):2936–2946. 16586430 10.1002/pmic.200500331

[ref43] Thomès L, Bojar D. The role of fucose-containing glycan motifs across taxonomic kingdoms. Front Mol Biosci. 2021:8:755577. 34631801 10.3389/fmolb.2021.755577 PMC8492980

[ref44] Tomek MB, Neumann L, Nimeth I, Koerdt A, Andesner P, Messner P, Mach L, Potempa JS, Schäffer C. The S-layer proteins of *Tannerella forsythia* are secreted* via* a type IX secretion system that is decoupled from protein *O*-glycosylation. Mol Oral Microbiol. 2014:29(6):307–320. 24943676 10.1111/omi.12062 PMC4232474

[ref45] Tomek MB, Maresch D, Windwarder M, Friedrich V, Janesch B, Fuchs K, Neumann L, Nimeth I, Zwickl NF, Dohm JC, et al. A general protein *O*-glycosylation gene cluster encodes the species-specific glycan of the oral pathogen Tannerella forsythia: *O*-glycan biosynthesis and immunological implications. Fron Microbiol. 2018:9:2008. 10.3389/fmicb.2018.02008 PMC6120980 30210478

[ref46] Tomek MB, Janesch B, Braun ML, Taschner M, Figl R, Grünwald-Gruber C, Coyne MJ, Blaukopf M, Altmann F, Kosma P, et al. A combination of structural, genetic, phenotypic and enzymatic analyses reveals the importance of a predicted fucosyltransferase to protein *O*-glycosylation in the *Bacteroidetes*. Biomol. 2021:11(12):1795. 10.3390/biom11121795 PMC8698959 34944439

[ref47] Tsuchiya S, Aoki NP, Shinmachi D, Matsubara M, Yamada I, Aoki-Kinoshita KF, Narimatsu H. Implementation of GlycanBuilder to draw a wide variety of ambiguous glycans. Carbohydr Res. 2017:445:104–116. 28525772 10.1016/j.carres.2017.04.015

[ref48] Varki A, Cummings RD, Aebi M, Packer NH, Seeberger PH, Esko JD, Stanley P, Hart G, Darvill A, Kinoshita T, et al. Symbol nomenclature for graphical representations of glycans. Glycobiology. 2015:25(12):1323–1324. 26543186 10.1093/glycob/cwv091 PMC4643639

[ref49] Vartoukian SR, Adamowska A, Lawlor M, Moazzez R, Dewhirst FE, Wade WG. In vitro cultivation of 'unculturable' oral bacteria, facilitated by community culture and media supplementation with siderophores. PLoS One. 2016:11(1):e0146926. 26764907 10.1371/journal.pone.0146926 PMC4713201

[ref50] Veith PD, Nor Muhammad NA, Dashper SG, Likic VA, Gorasia DG, Chen D, Byrne SJ, Catmull DV, Reynolds EC. Protein substrates of a novel secretion system are numerous in the *Bacteroidetes* phylum and have in common a cleavable C-terminal secretion signal, extensive post-translational modification, and cell-surface attachment. J Proteome Res. 2013:12(10):4449–4461. 24007199 10.1021/pr400487b

[ref51] Veith PD, Scott NE, Reynolds EC. Characterization of the *O*-glycoproteome of *Tannerella forsythia*. mSphere. 2021:6(5):e0064921. 34523981 10.1128/mSphere.00649-21 PMC8550257

[ref52] Veith PD, Shoji M, Scott NE, Reynolds EC. Characterization of the *O*-glycoproteome of *Porphyromonas gingivalis*. Microbiol Spectr. 2022:10(1):e0150221. 34985300 10.1128/spectrum.01502-21 PMC8729774

[ref53] Vinogradov E, Perry MB, Kay WW. The structure of the glycopeptides from the fish pathogen *Flavobacterium columnare*. Carbohydr Res. 2003:338(23):2653–2658. 14670723 10.1016/j.carres.2003.08.012

[ref54] Watanabe Y, Aoki-Kinoshita KF, Ishihama Y, Okuda S. GlycoPOST realizes FAIR principles for glycomics mass spectrometry data. Nucleic Acids Res. 2020:49(D1):D1523–D1528. 10.1093/nar/gkaa1012 PMC7778884 33174597

[ref55] Wessel D, Flügge U. A method for the quantitative recovery of protein in dilute solution in the presence of detergents and lipids. Anal Biochem. 1984:138(1):141–143. 6731838 10.1016/0003-2697(84)90782-6

[ref56] Ye X, Paul B, Mo J, Reynolds EC, Ghosal D, Veith PD. Ultrastructural and glycoproteomic characterization of *Prevotella intermedia*: insights into *O*-glycosylation and outer membrane vesicles. Microbiology. 2024:13(2):e1401. 10.1002/mbo3.1401 PMC10897501 38409911

[ref57] Zarschler K, Janesch B, Pabst M, Altmann F, Messner P, Schäffer C. Protein tyrosine *O*-glycosylation -a rather unexplored prokaryotic glycosylation system. Glycobiology. 2010:20(6):787–798. 20200052 10.1093/glycob/cwq035 PMC4397588

[ref58] Zwickl NF, Stralis-Pavese N, Schäffer C, Dohm JC, Himmelbauer H. Comparative genome characterization of the periodontal pathogen *Tannerella forsythia*. BMC Genomics. 2020:21(1):150. 32046654 10.1186/s12864-020-6535-y PMC7014623

