# Supplementary material for: Deciphering fucosylated protein-linked O-glycans in oral Tannerella serpentiformis: Insights from NMR spectroscopy and glycoproteomics
Source: Glycobiology. 2024 Sep 19;34(12):cwae072. doi: 10.1093/glycob/cwae072 (PMC11632369; doi:10.1093/glycob/cwae072)
Supplement: SI_GLYCO-2024-00072_13092024_cwae072 [file si_glyco-2024-00072_13092024_cwae072.pdf]

Supplementary Information

Deciphering Fucosylated Protein-Linked *O*-Glycans in Oral *Tannerella serpentiformis*: Insights from NMR Spectroscopy and Glycoproteomics

Stephanie Walcher[1], Fiona F. Hager-Mair[1], Johannes Stadlmann[1], Hanspeter Kählig[2] and Christina Schäffer[1,*]

[1]Institute of Biochemistry, Department of Chemistry, University of Natural Resources and Life Sciences, Vienna, Austria and [2]Department of Organic Chemistry, Faculty of Chemistry, University of Vienna, Vienna, Austria

*To whom correspondence should be addressed; e-mail: christina.schaeffer@boku.ac.at

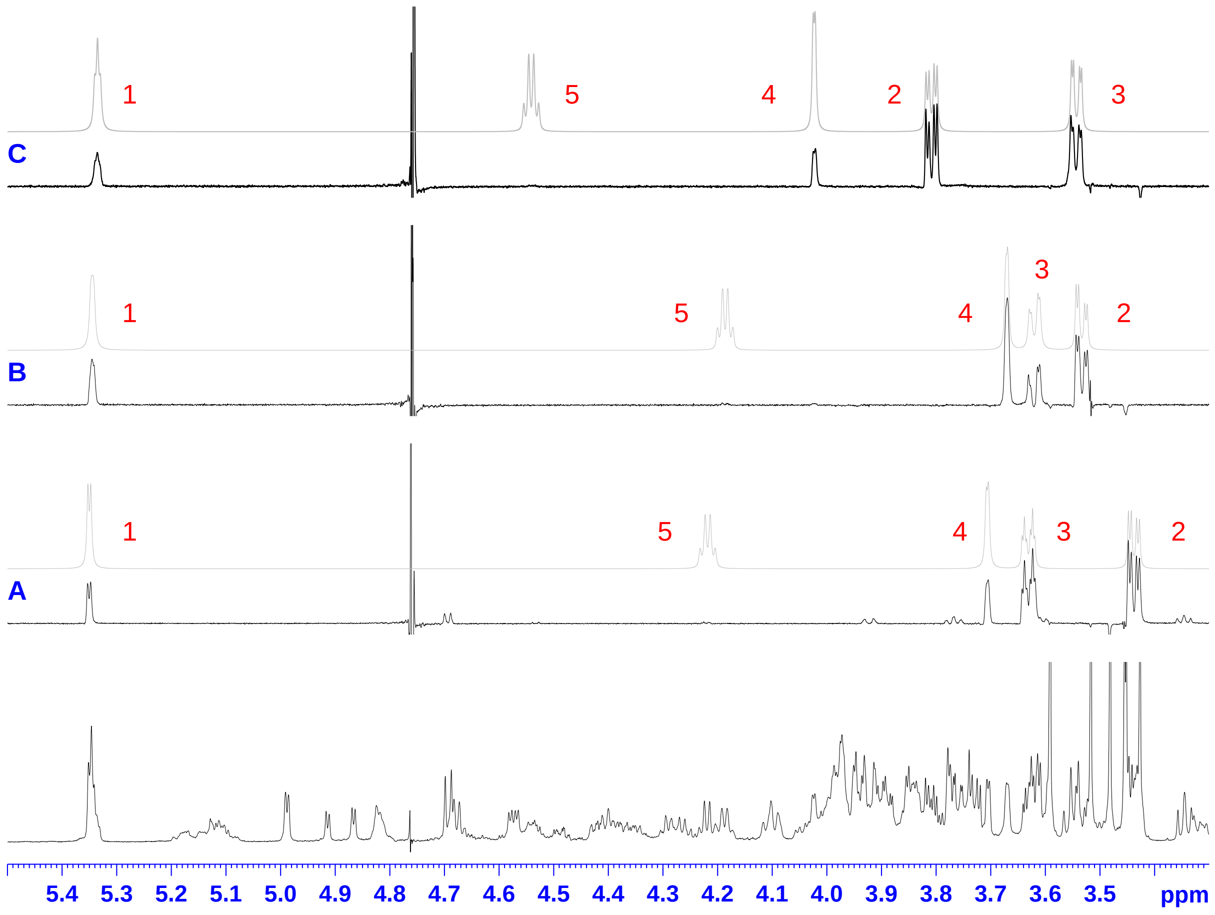

Supplementary Fig. S1. Spin systems of fucoses **A, B**, and **C** of the purified protein-linked *O*-glycan from *T. serpentiformis*, derived from DREAMTIME selection of protons 2 and 3 followed by TOCSY spin-lock. Gray traces, calculated spectra derived from spin-simulation, protons 6 not shown. Lower trace, ^{1}H NMR spectrum.

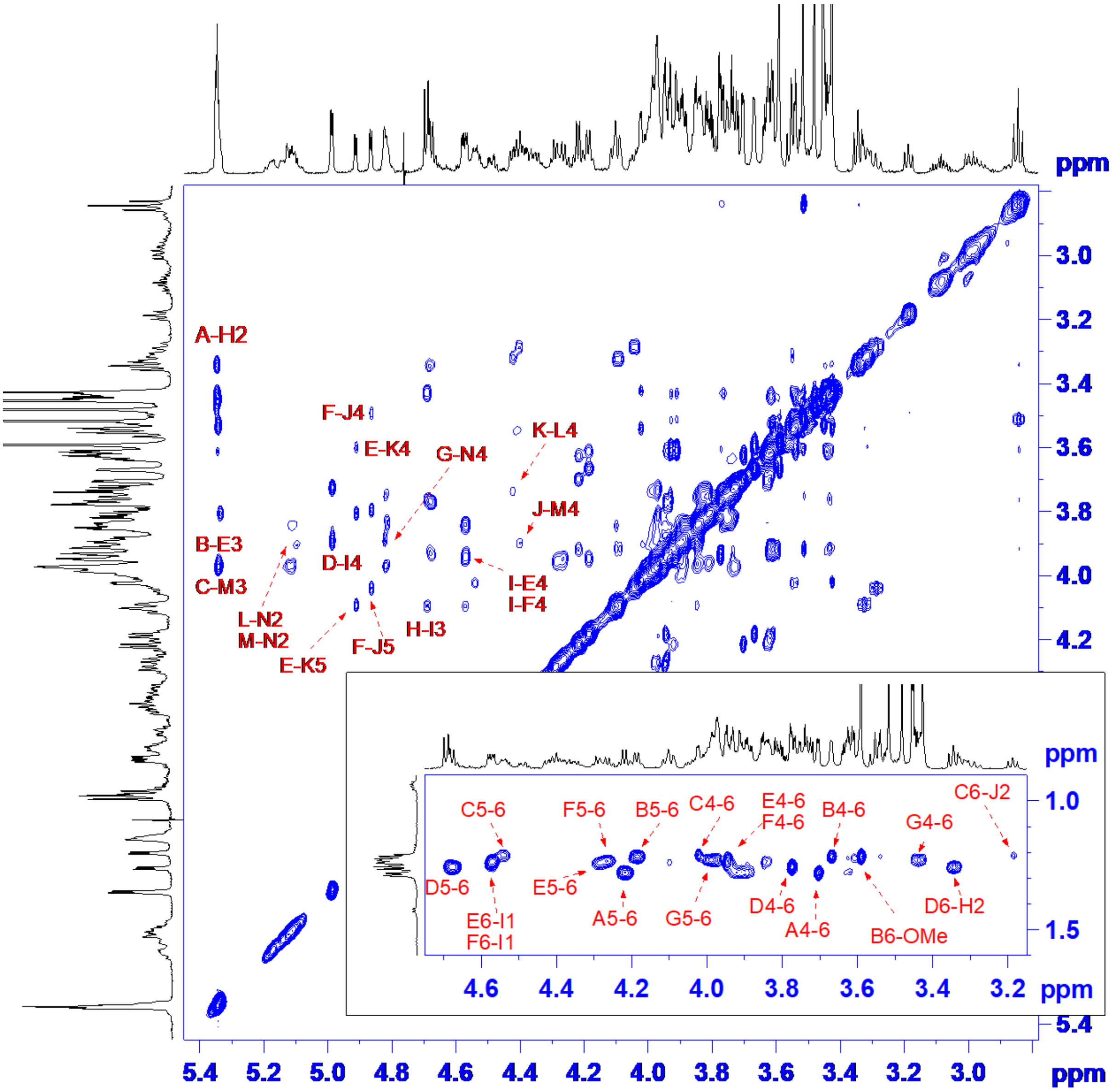

Supplementary Fig. S2. NOESY spectrum of the purified protein-linked *O*-glycan from *T. serpentiformis*, with annotation of the inter residue cross peaks starting from the anomeric protons. Inset, region for the 6-deoxy sugars showing cross peaks starting from the methyl groups to either H5 or H4 within the saccharides, and two inter residue cross peaks (**D**-H6 to **H**-H2, **C**-H6 to **J**-H2), respectively. Left and upper traces, ^{1}H NMR spectrum.

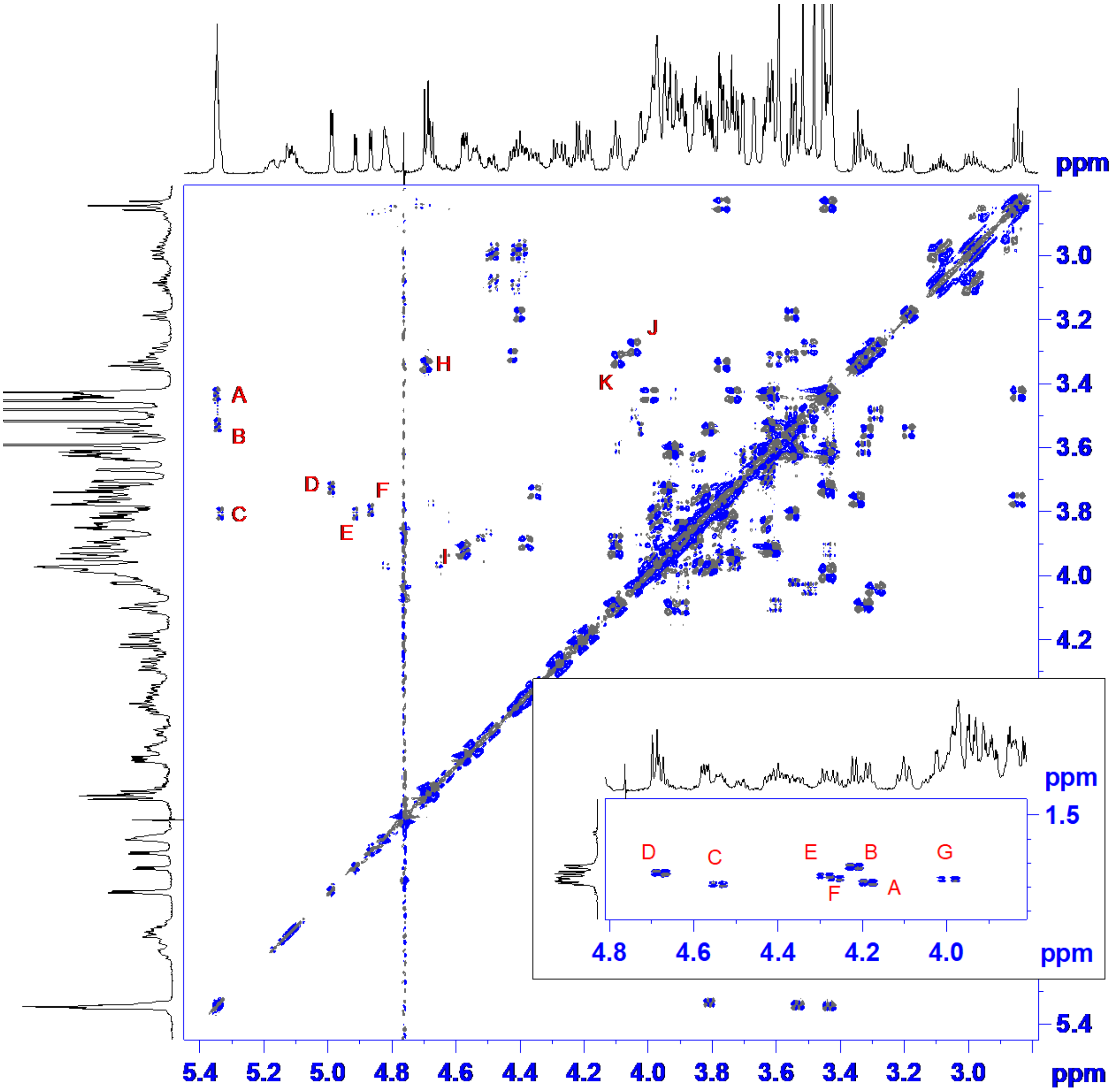

Supplementary Fig. S3. DQF-COSY spectrum of the purified protein-linked *O*-glycan from *T. serpentiformis*, with annotation of the cross peaks between H1 and H2 of the individual saccharides. Inset, region for the 6-deoxy sugars showing cross peaks between the methyl groups 6 and H5. Left and upper traces, ^{1}H NMR spectrum.

.

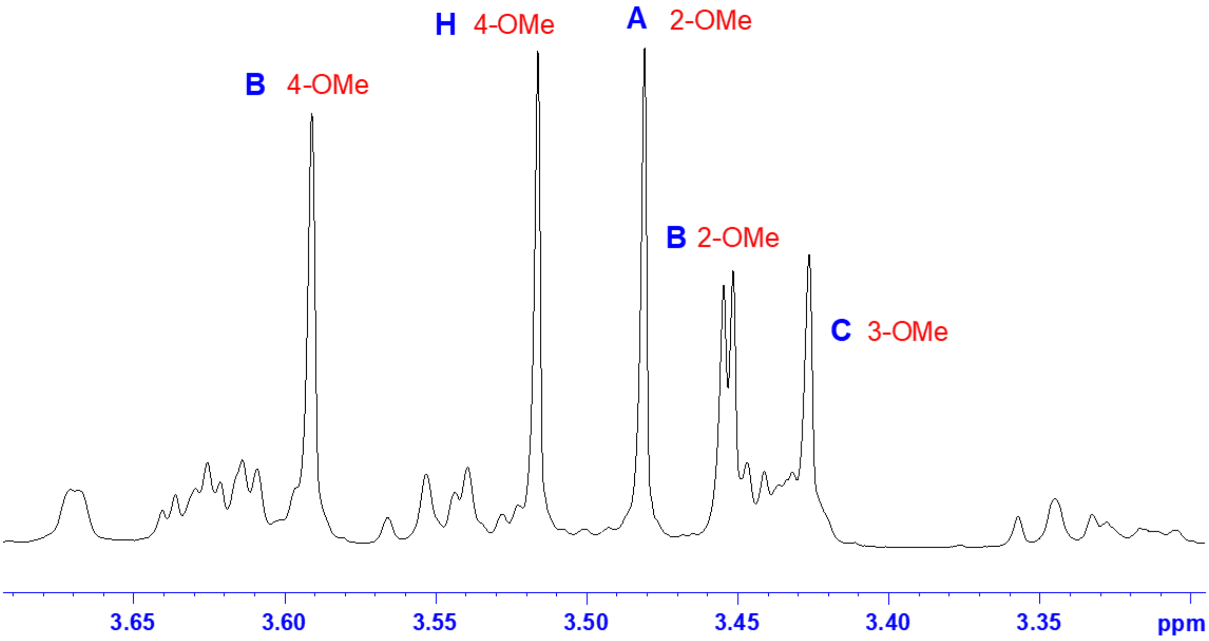

Supplementary Fig. S4. Region of ^{1}H NMR spectrum of the purified protein-linked *O*-glycan from *T. serpentiformis* showing the methoxy signals.

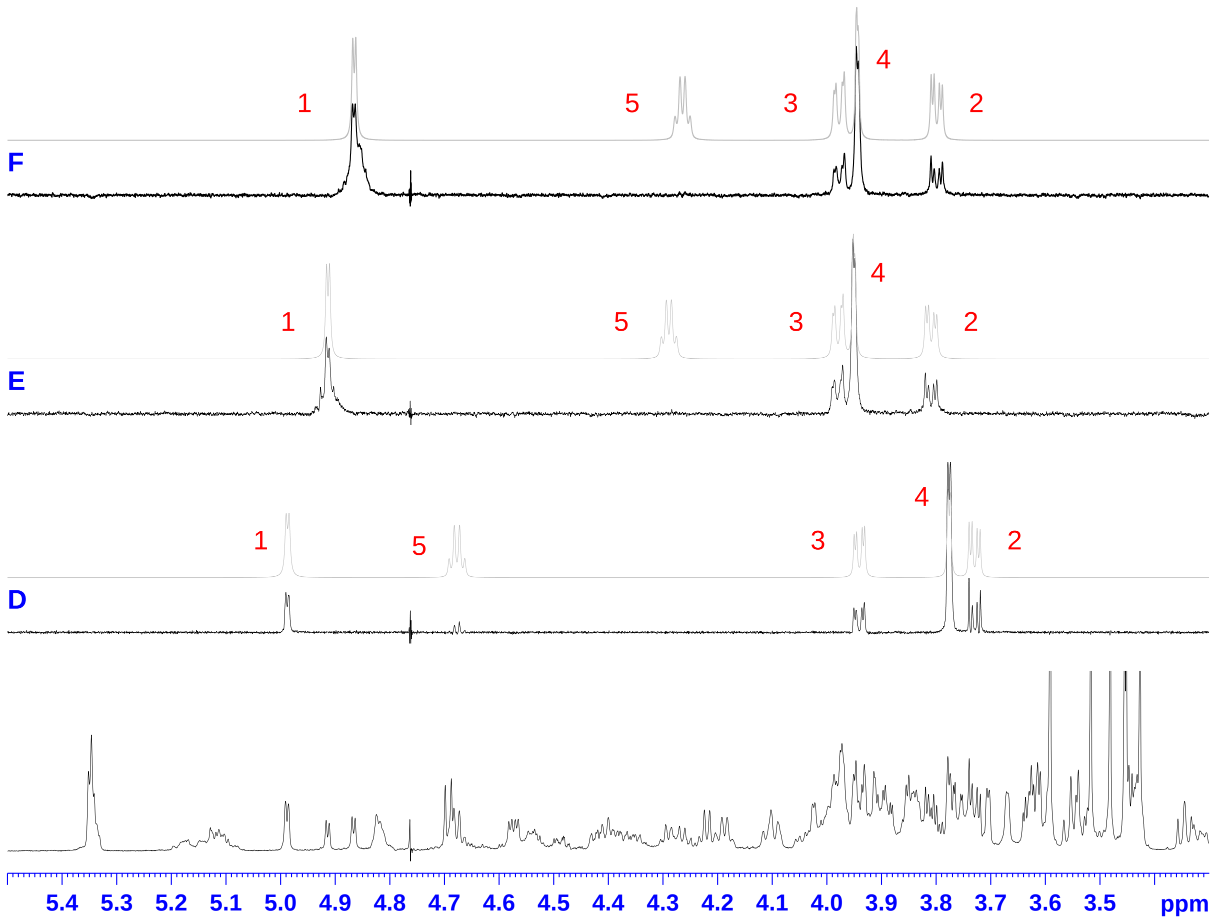

Supplementary Fig. S5. Spin systems of fucoses **D**/**E**/**F** of the purified protein-linked *O*-glycan from *T. serpentiformis*, derived from 1D-TOCSY experiments selecting the anomeric proton. Gray traces, calculated spectra derived from spin-simulation, protons 6 not shown. Lower trace, ^{1}H NMR spectrum.

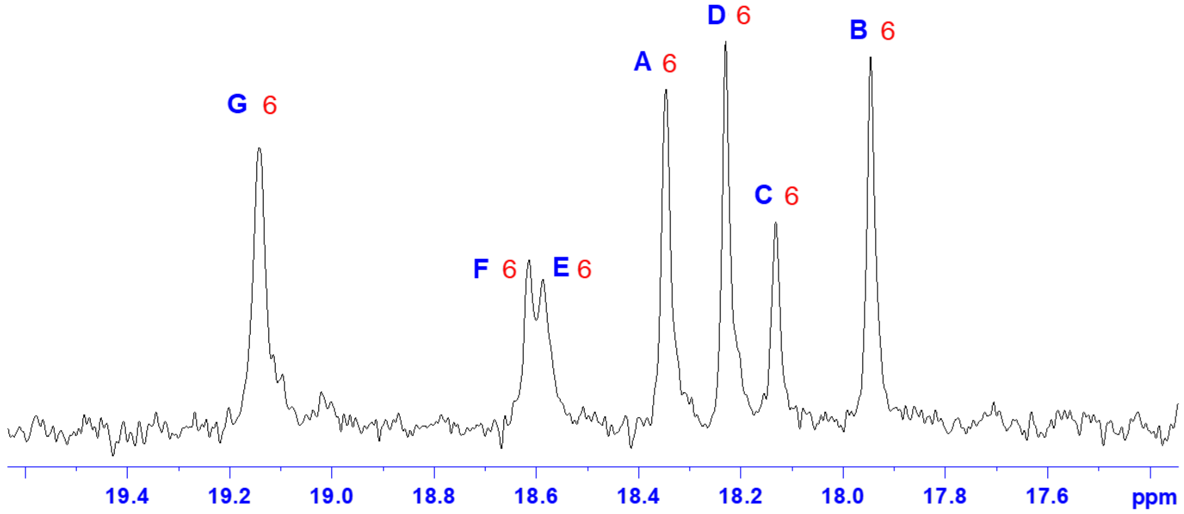

Supplementary Fig. S6. Region of ^{13}C NMR spectrum of the purified protein-linked *O*-glycan from *T. serpentiformis* showing the methyl signals of the 6-deoxy sugars.

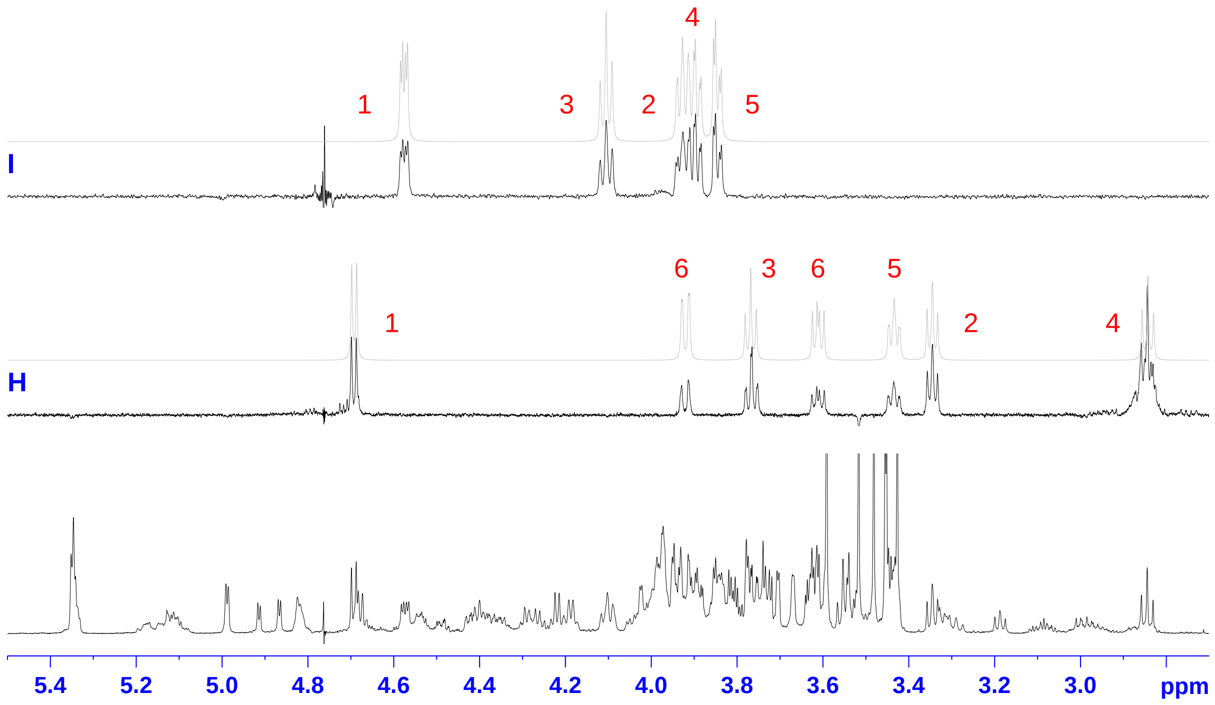

Supplementary Fig. S7. Spin systems of the glucose type sugars **H** and **I** of the purified protein-linked *O*-glycan from *T. serpentiformis*, derived from 1D-TOCSY experiments selecting proton 4 (sugar **H**) or from DREAMTIME selection of protons 1 and 2 followed by TOCSY spin-lock (sugar **I**). Gray traces, calculated spectra derived from spin-simulation. Lower trace, ^{1}H NMR spectrum.

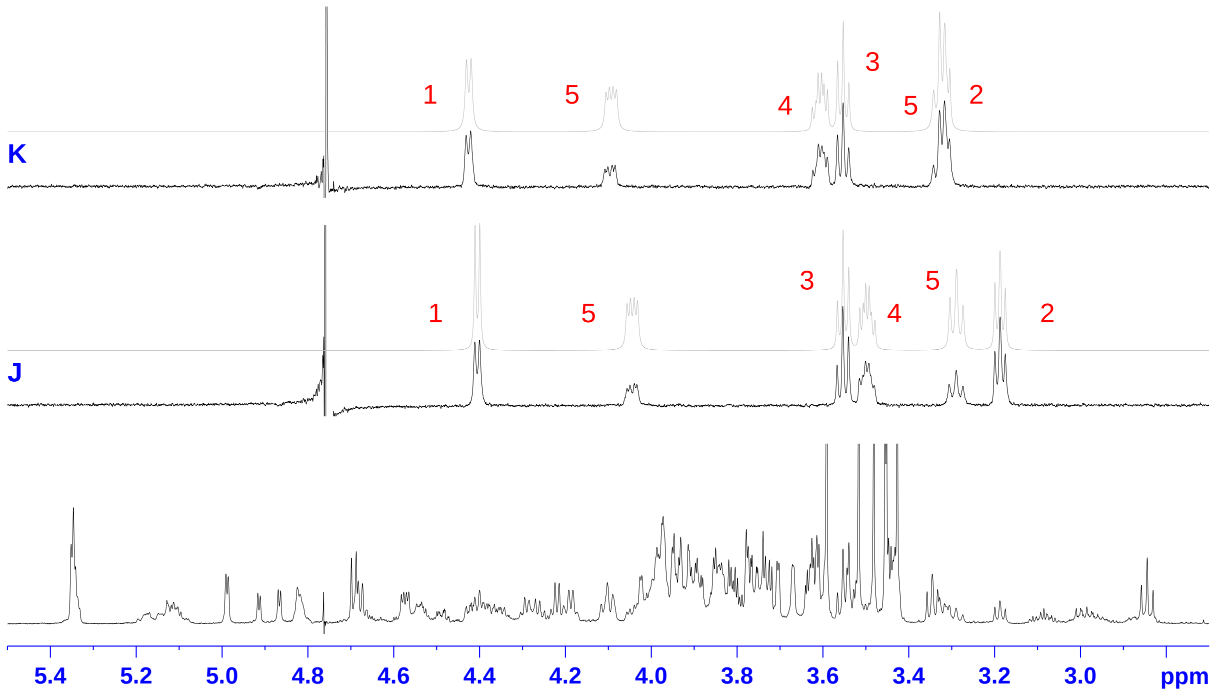

Supplementary Fig. S8. Spin systems of the xyloses **J** and **K** of the purified protein-linked *O*-glycan from *T. serpentiformis*, derived from DREAMTIME selection of protons 1 and 2 followed by TOCSY spin-lock. Gray traces, calculated spectra derived from spin-simulation. Lower trace, ^{1}H NMR spectrum.

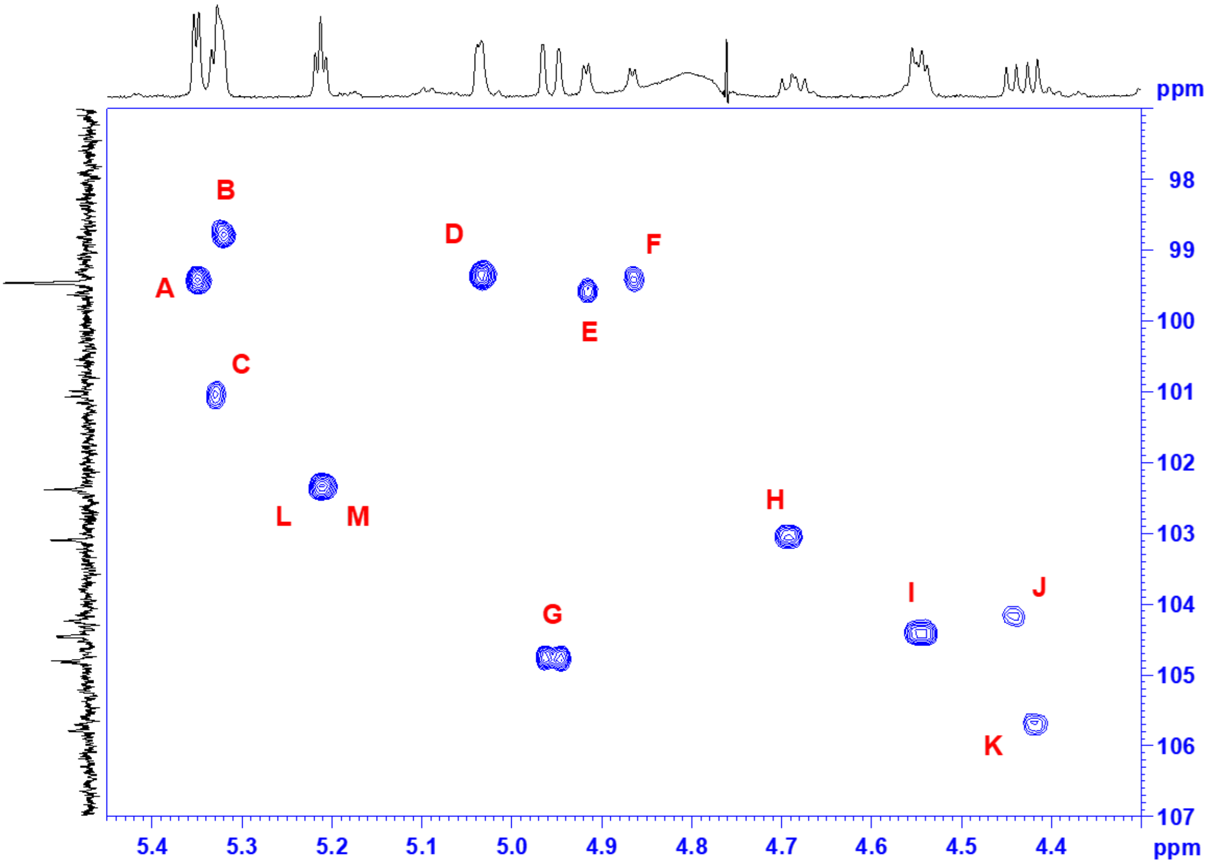

Supplementary Fig. S9. HSQC spectrum of the β-eliminated *O*-glycan from *T. serpentiformis*, section showing the anomeric region. Top trace, ^{1}H NMR spectrum; left trace, ^{13}C NMR spectrum.

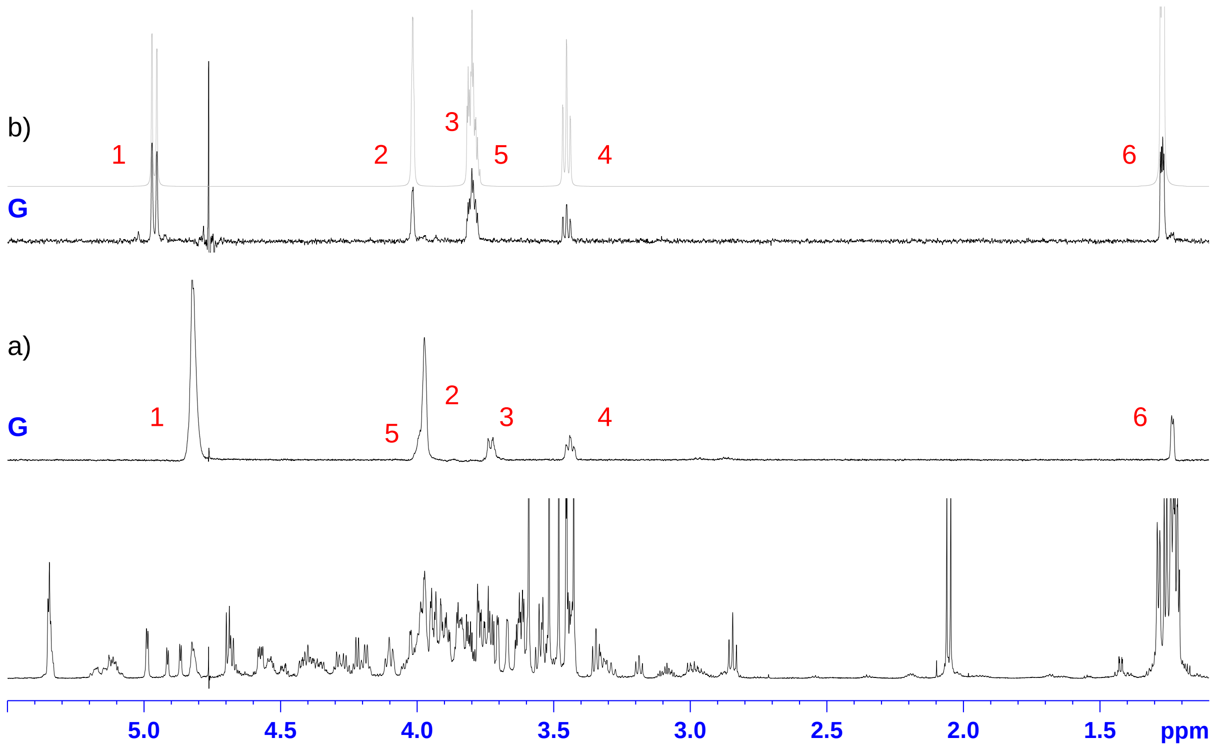

Supplementary Fig. S10. Spin systems of the rhamnose **G** of the purified protein-linked *O*-glycan from *T. serpentiformis* (trace a)) and of the β-eliminated glycan (trace b)), derived either from a 1D-TOCSY experiment selecting proton 1, (trace a)), or DREAMTIME selection of protons 2 and 3 followed by TOCSY spin-lock (trace b)). Gray trace, calculated spectrum derived from spin-simulation. Lower trace, ^{1}H NMR spectrum.

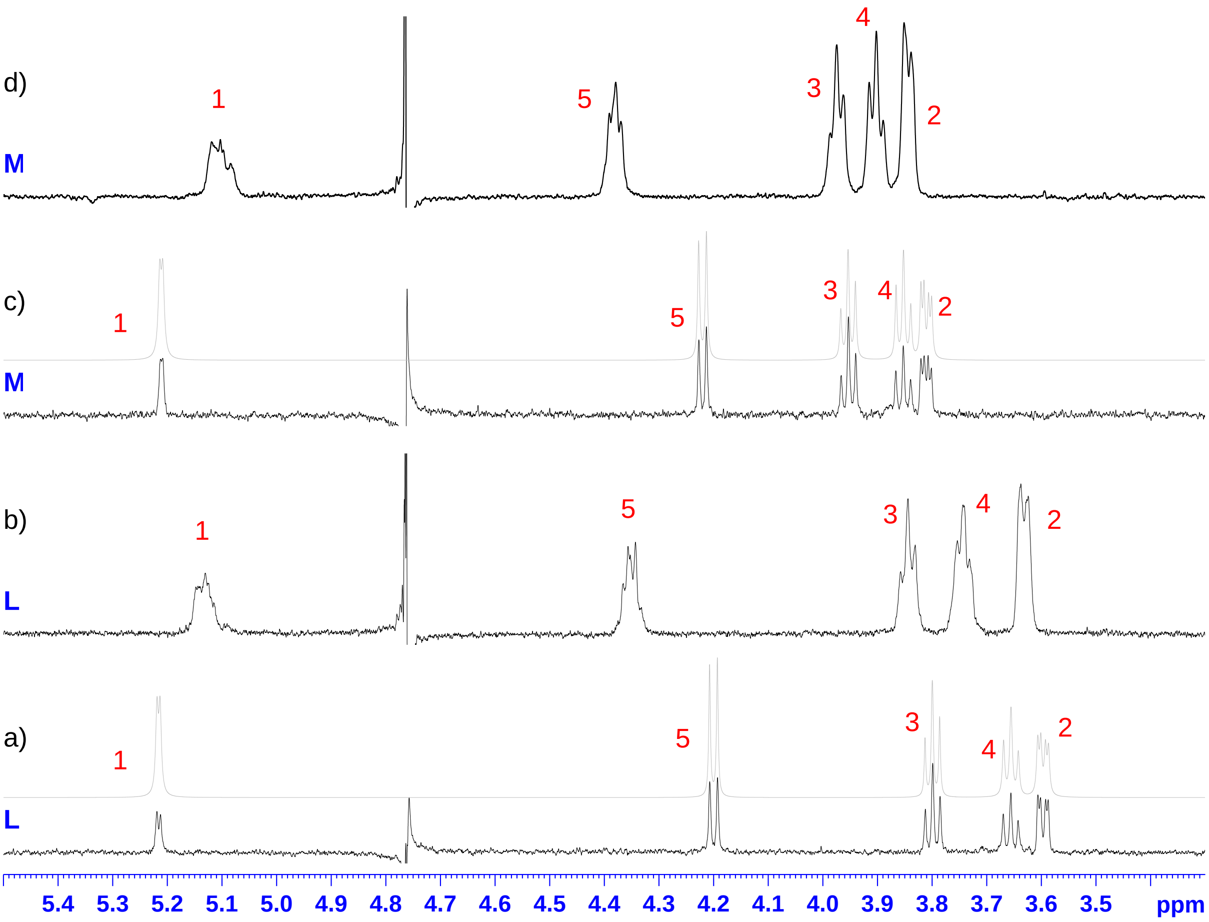

Supplementary Fig. S11. Spin systems of the glucuronic acids **L** and **M** of the purified protein-linked *O*-glycan from *T. serpentiformis* (trace a) and c)) and of the β-eliminated glycan (trace b) and d)), derived from DREAMTIME selection of protons 4 and 5 followed by TOCSY spin-lock. Gray traces, calculated spectra derived from spin-simulation.

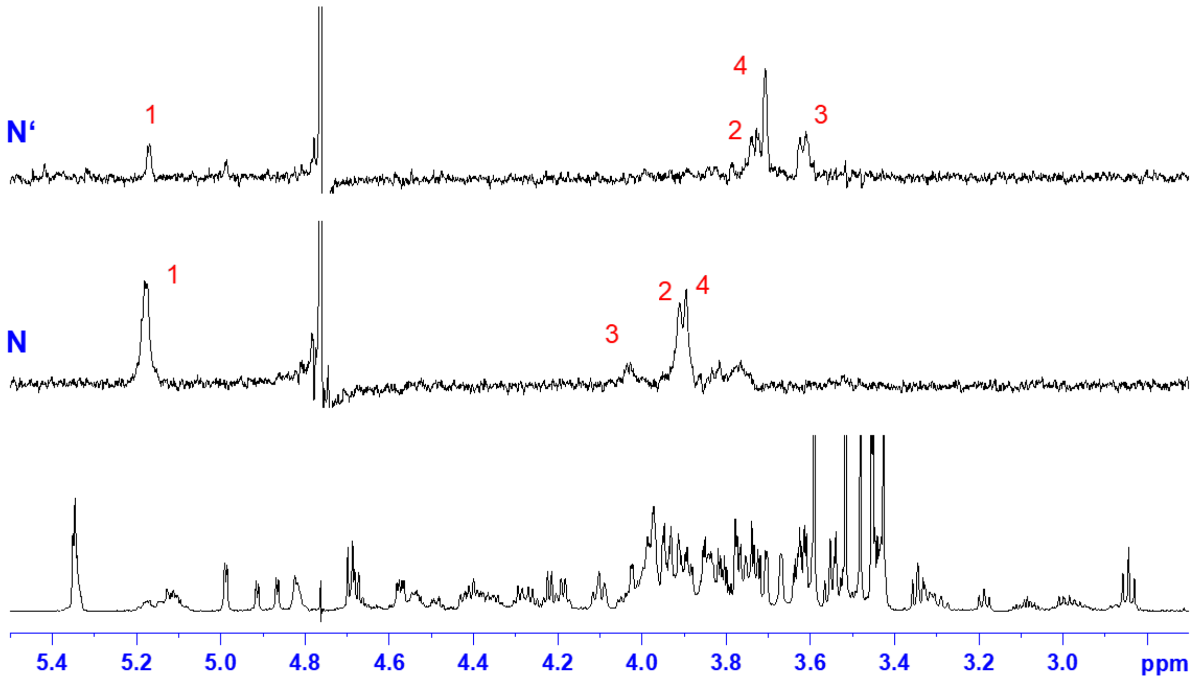

Supplementary Fig. S12. Spin systems of galactose **N**/**N**´ of the purified protein-linked *O*-glycan from *T. serpentiformis*, derived from DREAMTIME selection of protons 1 and 2 followed by TOCSY spin-lock. Lower trace, ^{1}H NMR spectrum.

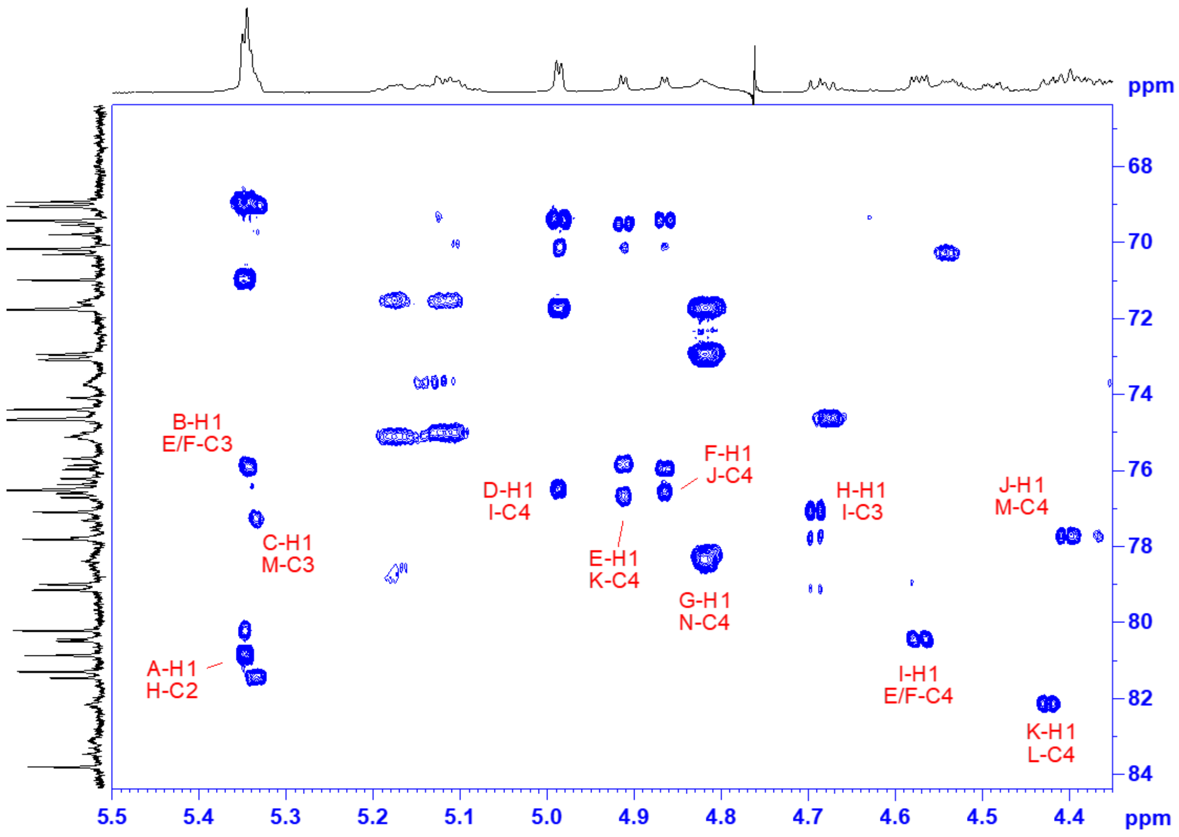

Supplementary Fig. S13. HMBC spectrum of the purified protein-linked *O*-glycan from *T. serpentiformis*, section showing the glycosidic linkage information. The cross peaks between the anomeric protons and the corresponding carbons of the linked monosaccharides are denoted. Top trace, ^{1}H NMR spectrum; left trace, ^{13}C NMR spectrum.

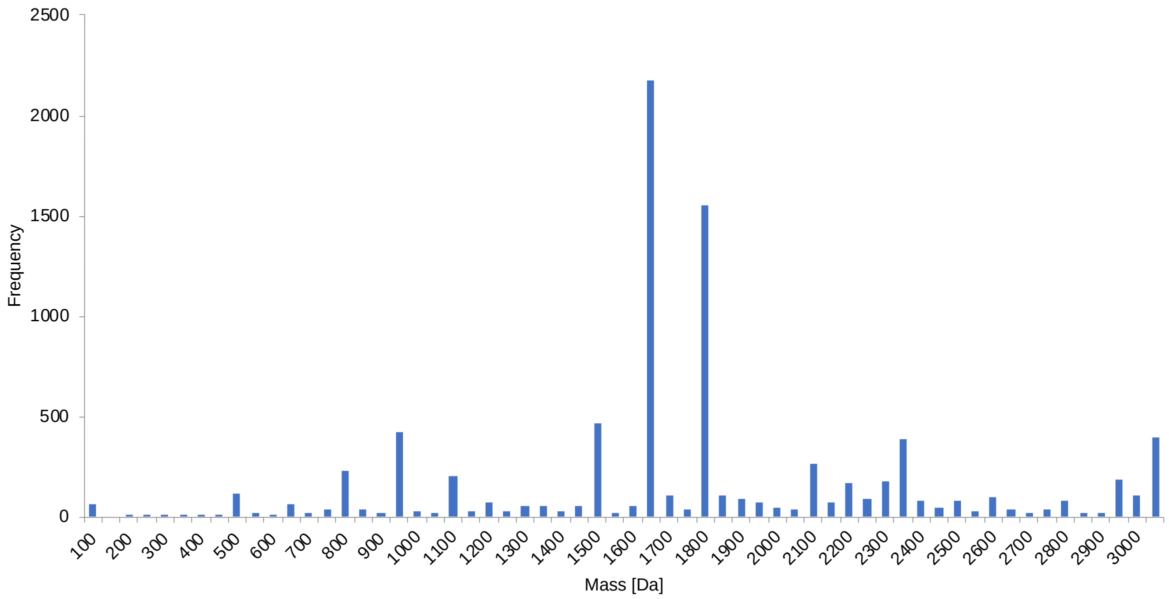

Supplementary Fig. S14. Glycan mass distribution in histogram form derived from a *T. serpentiformis* whole-cell protein sample, where the two main peaks at ~1,650 Da and ~1,800 Da correspond to the two main glycoforms G1 (decasaccharide, 1,634 Da) and G2 (undecasaccharide, 1,794 Da), differing in one *O*-methylated Fuc residue. Compare with Fig. 3.

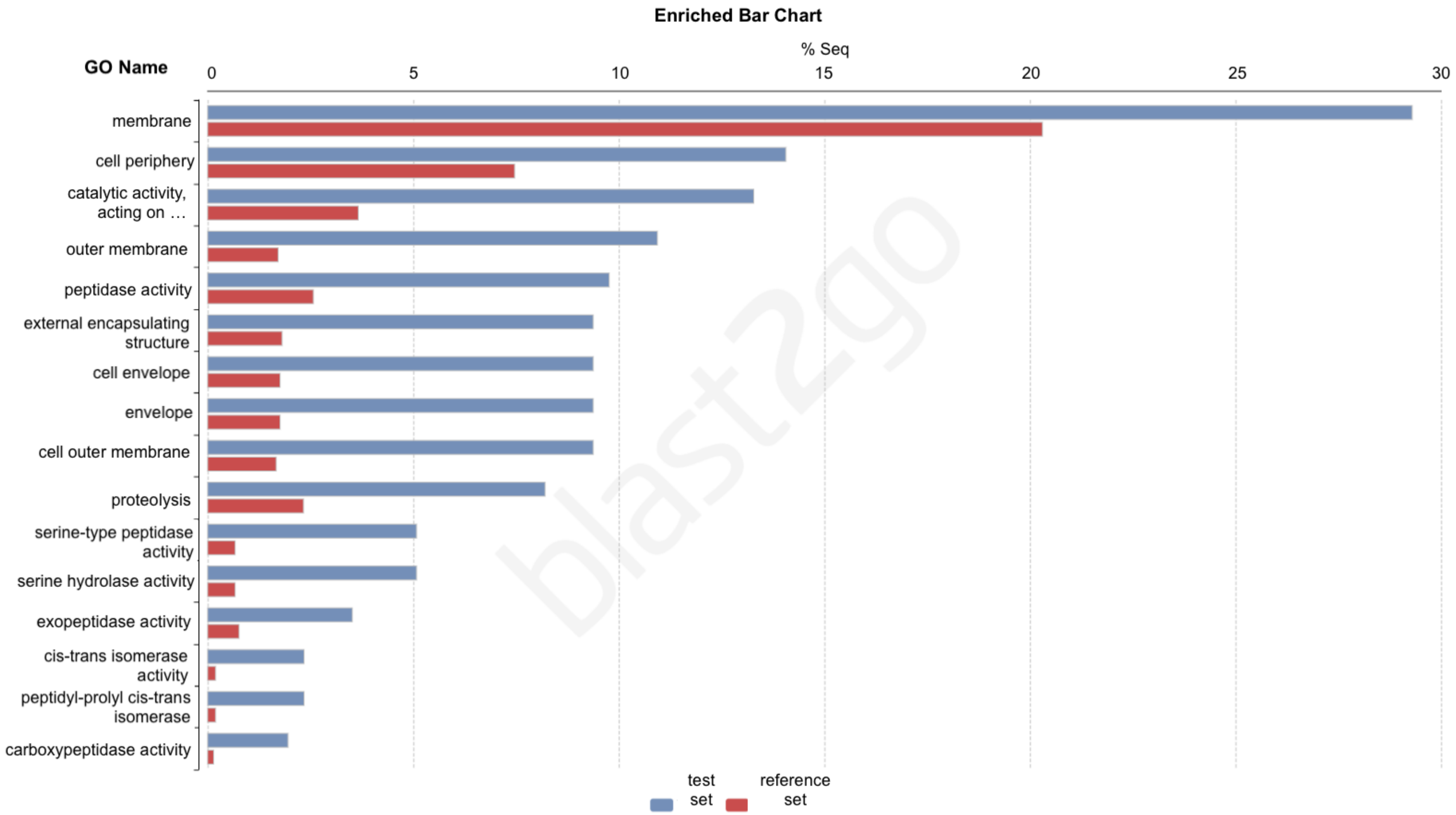

Supplementary Fig. S15. GO-term enrichment analysis (Fisher's Exact Test) was performed for glycoproteins (*i.e.*, test set; blue bars) *versus* all protein sequences identified in this study, as species specific reference sets (red bars), using Blast2Go (version 6.0.3 - build 202109151544) (Götz et al. 2008). Fonts edited in Preview (Version 11.0 – build 1056.2.4).

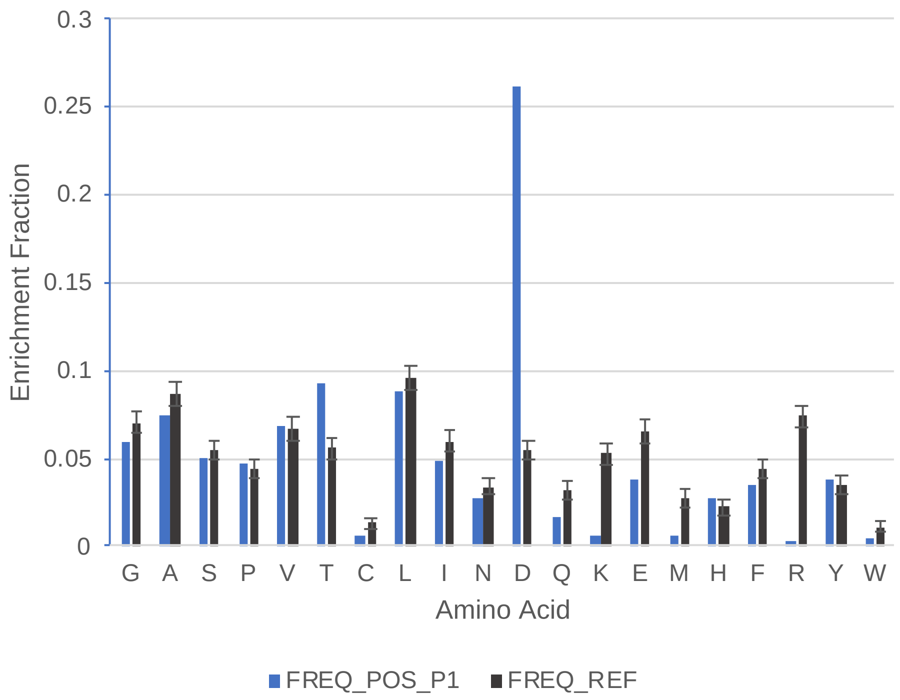

Supplementary Fig. S16. Enriched amino acids in the first position of the amino acid motif (X)(S/T)(X) in glycopeptides derived from whole *T. serpentiformis* cells as determined against an unglycosylated proteome background (UP000240373). Only "D" and to a minor degree "T" are statistically significant candidates. Figure generated with IceLogo (Colaert et al. 2009).

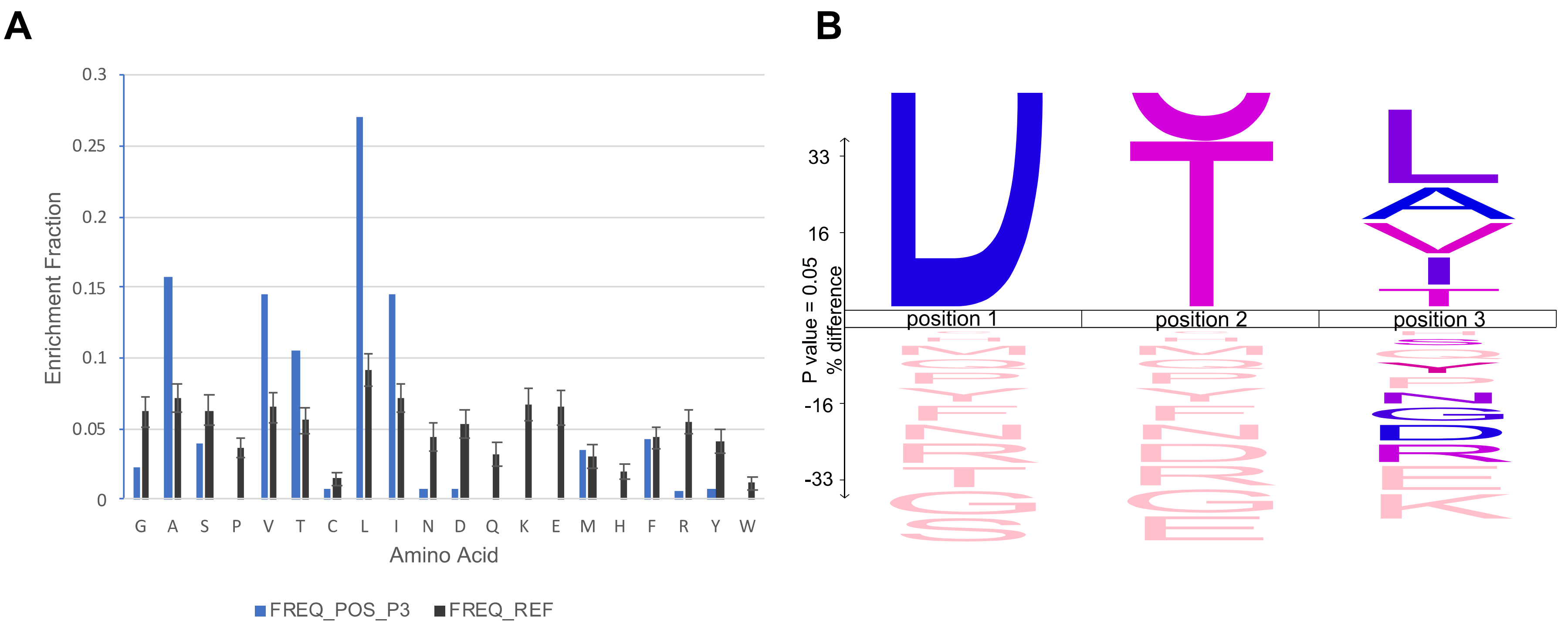

Supplementary Fig. S17. **A)** Enriched amino acids in the third position of the amino acid motif (D)(S/T)(X) in glycopeptides derived from *T. forsythia* cells (see Supplementary Information provided by Veith et al. 2021) as determined against an unglycosylated proteome background (taxon 28112, UP000182057). **B)** General amino acid sequence motif (D)(S/T)(L/V/T/A) based on identified glycopeptides (Veith et al. 2021) with the motif search D(S/T)(X). The positions are occupied equally as in *T. serpentiformis* (compare to Fig. 5). All figures were generated with IceLogo (Colaert et al. 2009).

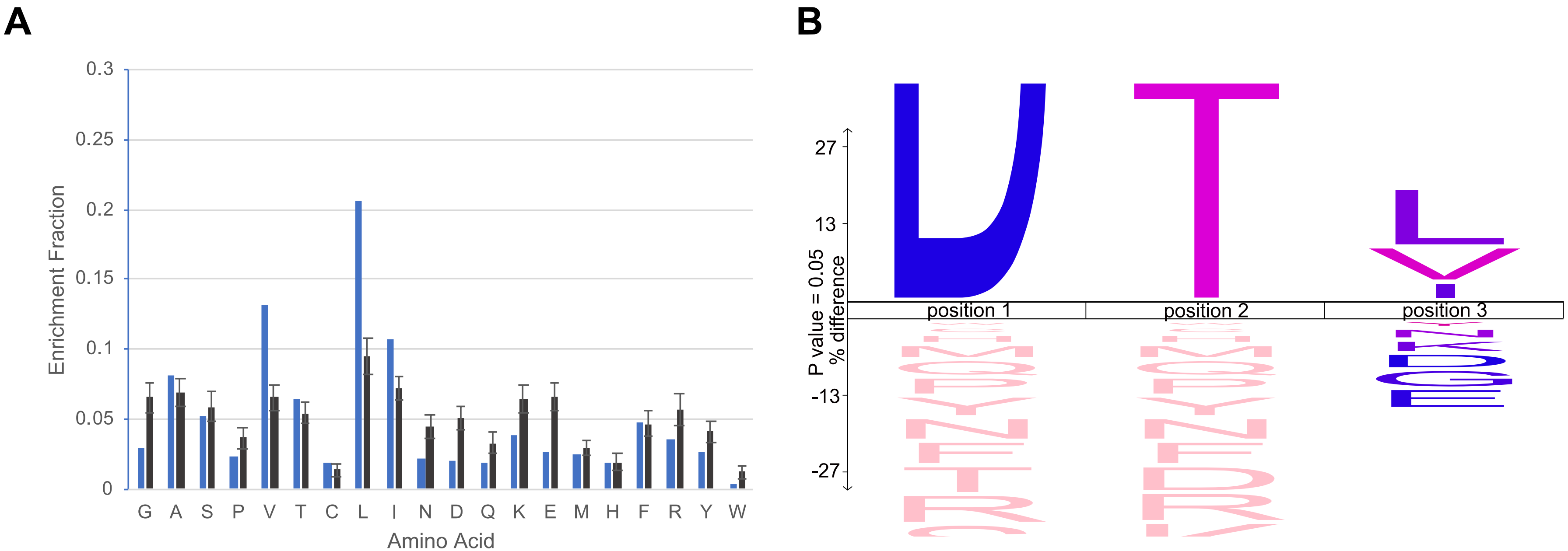

Supplementary Fig. S18: **A)** Enriched amino acids in the third position of the amino acid motif (D)(S/T)(X) in full glycoprotein sequences retrieved from the proteome UP000182057 of *T. forsythia* as determined against an unglycosylated proteome background. **B)** General amino acid sequence motif (D)(S/T)(L/V/I) based on full glycoprotein sequences retrieved from the *T. serpentiformis* proteome (UP000240373) with the motif (D)(S/T)(X). Compared to the glycopeptide search, where (L/V/T/A) were significant candidates, only (L/V/I) remain statistically significant. All figures were generated with IceLogo (Colaert et al. 2009).

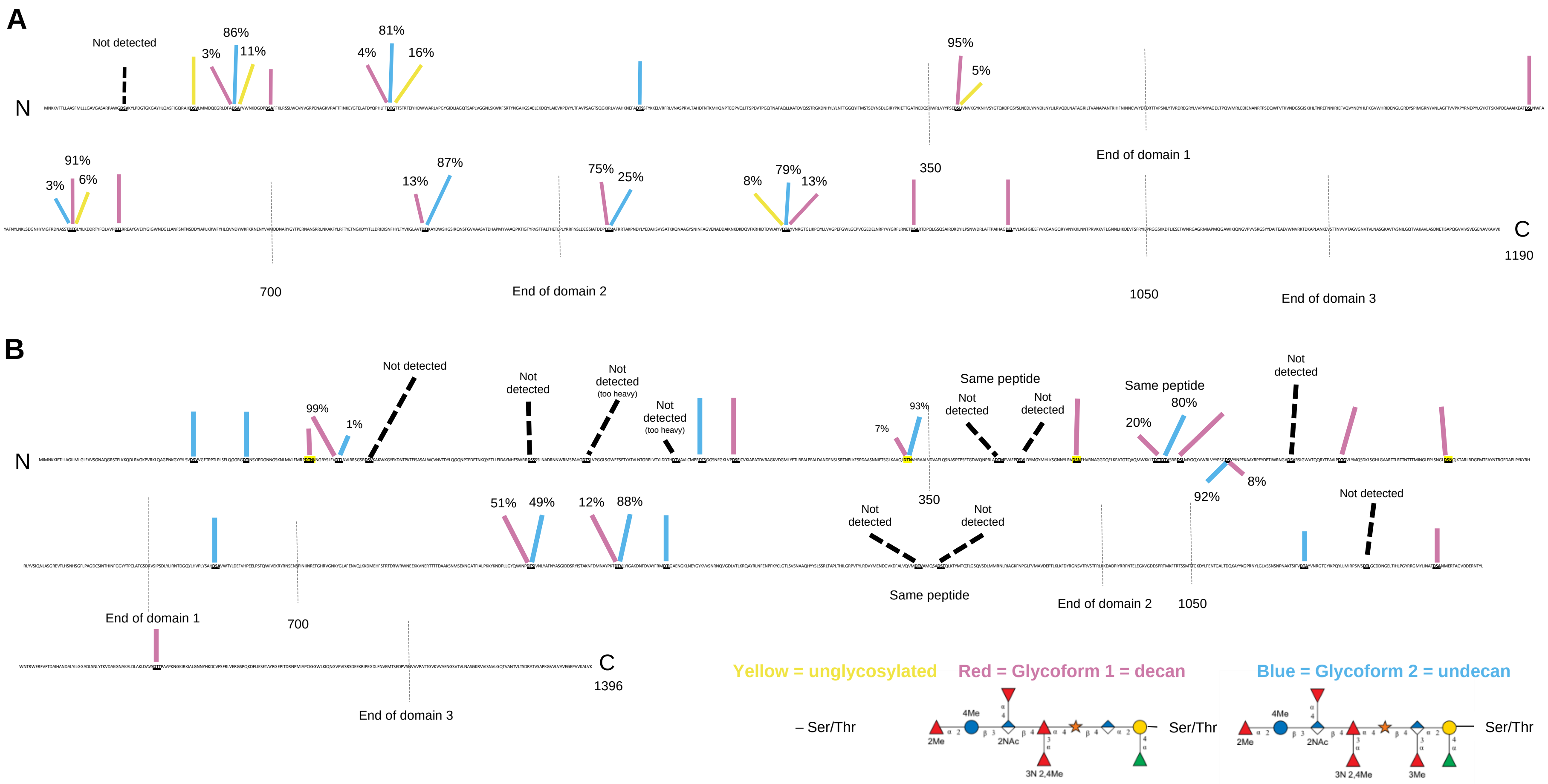

Supplementary Fig. S19. Linear distribution of *T. serpentiformis* glycoforms G1 and G2 over the primary sequence of the S-layer proteins. **A)** TssA (BCB71_RS00675 GenBank: CP017038.2, A0A2R4KII9) and **B)** TssB (BCB71_RS00680 GenBank: CP017038.2, A0A2R4KIF3). Glycoforms 1 and 2 are marked in red and blue and unglycosylated sites are marked in yellow.

Supplementary Table S1

Identified glycopeptide sequences derived from cut-out SDS-PAGE bands at the expected positions for the two S-layer proteins, A0A2R4KII9 (TssA) and A0A2R4KIF3 (TssB). Eleven unique UniProt sequences were identified, including the two S-layer proteins.

Peptide Sequence	UniProt ID
DSSSSLNADR	A0A2R4KIF3
AAQLDTNVHR	A0A2R4KIF3
LVYYPSGDSVYINPFK	A0A2R4KIF3
TSIFVDTAYVNR	A0A2R4KIF3
NEFADTSSFYKK	A0A2R4KII9
LDFADSAYVWNKDGDPDSAFFK	A0A2R4KII9
LVYYPSEDSLIVNVK	A0A2R4KII9
LTDTTDTVSR	A0A2R4KIF3
YSLFVDTLNVIR	A0A2R4KIF3
RFNSLDEGSIATDDPDTVAFR	A0A2R4KII9
LDAVSDTTPAAPK	A0A2R4KIF3
TYFQLVVPDTLRR	A0A2R4KII9
TDTVLYIGAK	A0A2R4KIF3
LAFTPAIHAGDTLYVLNGHSIEEFYVK	A0A2R4KII9
NEFADTSSFYK	A0A2R4KII9
TGAHFNITADTTLFAVWAIDK	A0A2R4KFB1
TGAHFNITADTTLFAVWAIDK	A0A2R4KIG3
KLTDTTDTVSR	A0A2R4KIF3
GDTINSYIPDGNNGSK	A0A2R4KIF3
FTSTHLNEDTTNIISR	A0A2R4KHV7
GLAVTDTIK	A0A2R4KII9
TDSLICHDSLIR	A0A2R4KHV7
TDSLICHDSLIR	A0A2R4KJL6
ADSLIFHSDFIK	A0A2R4KJC3
RDSSSSLNADR	A0A2R4KIF3
VDSNFHVR	A0A2R4KIF3
RGNIHFFDSVR	A0A2R4KJC3
DLLQDDSVR	A0A2R4KFF8
LNTVIFDHTVLTCLTDSFDHVK	A0A2R4KJC3
AIKDSVLMMDQEGR	A0A2R4KII9
LDFADSAYVWNK	A0A2R4KII9
EATDSLNWFAYAFNYLNK	A0A2R4KII9
QPGGTFAITADTTLYAVWAVDK	A0A2R4KFB1
QPGGTFAITADTTLYAVWAVDK	A0A2R4KIG3
HIDTDWAIYVDTAYVNR	A0A2R4KII9
GQNFTITQDTTLFAVWGEDK	A0A2R4KFB1

GQNFTITQDTTLFAVWGEDK	A0A2R4KIG3
QNVMVVDHTVISFLTDSFDHVK	A0A2R4KHV7
LQFHTTSVDSIAEAFSGTGK	A0A2R4KHV7
CGNEGDSILHNR	A0A2R4KJC3
DTSGGSNFGK	A0A2R4KIF3
LVPDSFICVK	A0A2R4KIF3
FNSLDEGSIATDDPDTVAFRR	A0A2R4KII9
YNNGENVTVKDSGSLAR	A0A2R4KFB1
YNNGENVTVKDSGSLAR	A0A2R4KIG3
TYFQLVVPDTLR	A0A2R4KII9
DNASSTDTGLYILK	A0A2R4KII9
EHETFTITTDTTLFAVWGK	A0A2R4KFB1
EHETFTITTDTTLFAVWGK	A0A2R4KIG3
FNSLDEGSIATDDPDTVAFR	A0A2R4KII9
NETDSARTDPQLGSQSAIR	A0A2R4KII9
DGDPDSAFFK	A0A2R4KII9
LVVAHKNEFADTSSFYK	A0A2R4KII9
EYGTELAFDYQPHLFTDTGTTSTR	A0A2R4KII9
DNASSTDTGLYILKDDR	A0A2R4KII9
VYTEFDTIKLPDDTAK	A0A2R4KGM1
LTDTTAVVTGK	A0A2R4KHT7
DFALVQVMDTVAMQSADSTQLK	A0A2R4KIF3
INTNDTLYIVNFGNNGTNTCAAK	A0A2R4KHV7
VETEVNDTVVLHAR	A0A2R4KJC3
GNIHFFDSVR	A0A2R4KJC3
RNENYVVMDDNAR	A0A2R4KII9
WFSDIANCR	A0A2R4KI78

Supplementary Table S2

Glycoproteome data of *T. serpentiformis*.

The Excel sheet can be accessed under https://glycopost.glycosmos.org/preview/21059169826698ecfac4458, PIN CODE: 4998, file name: TannerellaHOT_GlycproteomeFinal.xlsx.

References

Colaert N, Helsens K, Martens L, Vandekerckhove J, Gevaert K. 2009. Improved visualization of protein consensus sequences by iceLogo. *Nat Methods.* 6(11):786-787. doi:10.1038/nmeth1109-786.

Götz S, García-Gómez JM, Terol J, Williams TD, Nagaraj SH, Nueda MJ, Robles M, Talón M, Dopazo J, Conesa A. 2008. High-throughput functional annotation and data mining with the Blast2GO suite. *Nucleic Acids Res.* 36(10):3420-3435. doi:10.1093/nar/gkn176.

Veith PD, Scott NE, Reynolds EC. 2021. Characterization of the *O*-glycoproteome of *Tannerella forsythia*. *mSphere.* e0064921. doi:10.1128/mSphere.00649-21.
